# Solid-State Structures and Properties of Lignin Hydrogenolysis Oil Compounds: Shedding a Unique Light on Lignin Valorization

**DOI:** 10.3390/ijms251910810

**Published:** 2024-10-08

**Authors:** Oliver J. Driscoll, Kristof Van Hecke, Christophe M. L. Vande Velde, Frank Blockhuys, Maarten Rubens, Tatsuhiro Kuwaba, Daniel J. van de Pas, Walter Eevers, Richard Vendamme, Elias Feghali

**Affiliations:** 1 Sustainable Polymer Technologies Team, Flemish Institute for Technological Research (Vito N.V.), Boeretang 200, 2400 Mol, Belgium; maarten.rubens@vito.be (M.R.); t.kuwaba@tue.nl (T.K.); walter.eevers@vito.be (W.E.); richard.vendamme@vito.be (R.V.); 2 New Zealand Forest Research Institute (Scion), Private Bag 3020, Rotorua 3046, New Zealand; daniel.vandepas@scionresearch.com; 3 XStruct, Department of Chemistry, Ghent University, Krijgslaan 281-S3, 9000 Ghent, Belgium; kristof.vanhecke@ugent.be; 4 iPRACS, Faculty of Applied Engineering, University of Antwerp, Groenenborgerlaan 171, 2020 Antwerp, Belgium; christophe.vandevelde@uantwerpen.be; 5 Department of Chemistry, University of Antwerp, Groenenborgerlaan 171, 2020 Antwerp, Belgium; frank.blockhuys@uantwerpen.be; 6 Chemical Engineering Program, Notre Dame University–Louaize, Zouk Mosbeh 1211, Lebanon

**Keywords:** lignin, single-crystal X-ray diffraction, lignin model, thermal properties, glycidylation, lignin epoxide, crystal structure, CLP intermolecular energy calculations

## Abstract

This article explores the important, and yet often overlooked, solid-state structures of selected bioaromatic compounds commonly found in lignin hydrogenolysis oil, a renewable bio-oil that holds great promise to substitute fossil-based aromatic molecules in a wide range of chemical and material industrial applications. At first, single-crystal X-ray diffraction (SCXRD) was applied to the lignin model compounds, dihydroconiferyl alcohol, propyl guaiacol, and eugenol dimers, in order to elucidate the fundamental molecular interactions present in such small lignin-derived polyols. Then, considering the potential use of these lignin-derived molecules as building blocks for polymer applications, structural analysis was also performed for two chemically modified model compounds, i.e., the methylene-bridging propyl-guaiacol dimer and propyl guaiacol and eugenol glycidyl ethers, which can be used as precursors in phenolic and epoxy resins, respectively, thus providing additional information on how the molecular packing is altered following chemical modifications. In addition to the expected H-bonding interactions, other interactions such as π–π stacking and C–H∙∙∙π were observed. This resulted in unexpected trends in the tendencies towards the crystallization of lignin compounds. This was further explored with the aid of DSC analysis and CLP intermolecular energy calculations, where the relationship between the major interactions observed in all the SCXRD solid-state structures and their physico-chemical properties were evaluated alongside other non-crystallizable lignin model compounds. Beyond lignin model compounds, our findings could also provide important insights into the solid-state structure and the molecular organization of more complex lignin fragments, paving the way to the more efficient design of lignin-based materials with improved properties for industrial applications or improving downstream processing of lignin oils in biorefining processes, such as in enhancing the separation and isolation of specific bioaromatic compounds).

## 1. Introduction

Society is disturbingly overdependent on non-renewable fossil fuel resources. With a growing global population, and, as a result, escalating demands on both material and energy, these resources are drastically diminishing. In response to this, and to alleviate the resulting significant environmental concerns, contemporary research has recently looked to more sustainable approaches, such as the circular bioeconomy and the biorefinery concept [1,2,3,4,5]. Lignocellulosic biomass is the most abundant biomass-derived, organic resource on Earth and will be at the core of these future sustainable approaches. There has been significant advancement in different biorefinery strategies and the fractionation of lignocellulose [6,7]. However, to make the more sustainable process economically viable, the current modern challenge for the scientific community has been the valorization of lignin; other than the cellulose and hemicellulose fractions, lignin is the last major constituent of wood that needs to be fully valorized for the lignocellulosic biorefinery process [5,6,7]. It is regarded as a low-value by-product, and often burnt as a cheap energy fuel, but there is a desire to convert this underutilized resource to more sustainable higher-value macromolecular materials and chemical applications (lignin-to-materials and lignin-to-chemicals) in a bid to aid lignin valorization [5,6,7,8,9,10,11,12].

Lignin is a structurally complex, cross-linked, amorphous, highly aromatic biopolymer with strong C–C and C–O linkages holding the three-dimensional network together. The most abundant linkage type is the β–O–4 ether moiety (~50–60% of all linkages depending on the source of lignin, plant species, and processing history) [6,7,9,10]. As a result of this high linkage abundance, and because C–O bonds are significantly weaker than condensed C–C bonds, the cleavage or scission of these ether linkages (β–O–4 and others such as α–O–4) can commonly be the target of lignin depolymerization methods using mild reaction conditions. Thus, this is a critical factor for the utilization of lignin for higher-value, well-defined chemicals and macromolecules, and its valorization [4,5,7,8,9,10].

As a result of the high structural complexity, the use of organic compounds as lignin models/mimics are commonly employed to aid analytical studies and improve our understanding of lignin’s structure and composition [13]. Single-crystal X-ray diffraction (SCXRD) is a powerful experimental technique in obtaining a definitive and complete, structural elucidation in three dimensions for a molecule or compound, i.e., the solid-state structure of the molecule or compound. This allows the observer to analyze aspects such as the intermolecular interactions and orientations of substituents at play [14]. Although the amorphous nature of lignin *prevents* SCXRD on actual lignin (native, technical, and depolymerized), there have been reports of studies on lignin models with a focus on helping to improve our knowledge of the biosynthetic pathway of lignin from the radical coupling of monolignols [15,16]. Notably, Lundquist et al. synthesized arylglycerol β-syringyl ether lignin model compounds, with β–O–4 ether linkages, and applied X-ray crystallography to study the stereochemistry and conformation which these lignin models adopt in an attempt to correlate the geometry and reactivity of the linkage [17,18,19]. Roblin et al. synthesized and evaluated a trimeric lignin model containing both β–O–4 ether and biphenyl, condensed 5–5′ linkages to study how the intermolecular interactions, such as hydrogen-bonding (H-bonding), controlled the conformation and aromatic ring orientations [16]. Despite lignin being amorphous, studies such as these show there is the possibility of organization between the monomeric units in the lignin polymeric network which may influence the biosynthesis and structure of the native lignin [16,20,21]. The important possible interactions at play in lignin (technical, native, and lignin-based materials), causing these organizations, are, for example, hydrophobic, π–π stacking and, in particular, H-bonding interactions [20,21,22].

While the crystallographic literature has focused on studying this monolignols-to-lignin biosynthetic route, there is seldom a focus on the lignin-to-materials synthetic route and, hence, the critical component related to lignin valorization [16,17,18,19]. Reductive catalytic fractionation (RCF), such as via metal-catalyzed hydrogenolysis, is a ‘lignin-first’ biorefinery approach that has recently gained attention as an attractive methodology. Superior lignin-derived polyol products can be obtained from native lignin via the breaking of the C–O ether linkages as mentioned earlier [4,5,7,8,9,10,23]. The resulting lignin hydrogenolysis oil (LHO) comprises a high concentration of monomer and dimer compounds, and oligomers with lower molecular weights, and the composition of these is dependent on the RCF catalytic system and wood source employed. Consequently, an overall enhancement in the properties of the lignin as a polyol can be achieved for future material synthesis [5,8].

**Figure 1:**
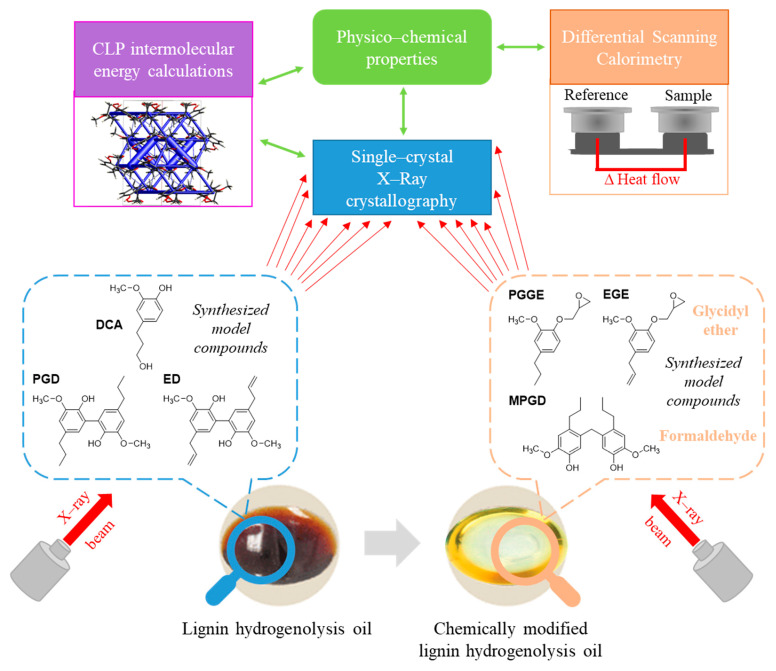
A general overview of this study where single-crystal X-ray crystallography (SCXRD) was applied to synthesized lignin model compounds commonly found in unmodified or chemically modified lignin hydrogenolysis oil. With the aid of differential scanning calorimetry (DSC) and Coulomb–London–Pauli (CLP) intermolecular energy calculations, the relationship between the SCXRD solid-state structures and their physico-chemical properties was evaluated alongside other non-crystallizable lignin model compounds.

In literature, there are limited reports on these LHO monomer and dimer compounds using SCXRD, and with even less focus on the compounds prominent in higher concentrations [24,25,26,27,28,29]. Epoxy resin polymers are central to the commercial thermoset materials employed globally in industry for applications such as coatings, electrical insulators or laminates, adhesives, electronic components, and composites [30,31,32,33,34]. Recently, with a desire to shift away from and replace the petrochemical curing agents commonly used with bio-derived ones, lignin has started to be explored as a precursor. This is because of its suitability as a reactive aromatic filler, the availability of large quantities at low cost for industry, the abundance of both reactive surface groups (hydroxy and carboxyl), and the various sources of lignin available. Depending on these sources and the variety of chemical modifications possible, the properties of the lignin can be tailored for a range of epoxy resin applications [31,32,33,34]. Interestingly, for lignin epoxy resin materials produced from these depolymerized LHOs, we previously reported the observation that semicrystalline structures with spherulite morphology were observed via scanning electron microscopy (SEM) and corroborated with dynamic mechanical temperature analysis (DMTA) [31]. We postulated that monomers or lower-molecular-weight components of the LHOs were responsible for the nucleation and growth of these crystallites. Hence, for these reasons, the primary focus of the current study was to apply SCXRD to *major* monomer and dimer compounds commonly present in depolymerized lignin hydrogenolysis oils (LHOs) in order to better understand and observe possible interactions, and molecular organizations, that would be present in LHO polyols before material synthesis (Figure 1). Improving our knowledge on the crystallization profile of model compounds and the subsequent interactions that would likely be present in the LHO could improve the downstream processing applications of lignin oils, such as either as polyols for material synthesis and/or enhancing the separation and isolation of specific bioaromatic compounds, both of which improve the lignin biorefining processes. As far as we are aware, this is the first time such a study has been attempted with SCXRD. This approach aligns with a recent study carried out by Johansson and co-workers, where the molecular structure and morphology (π–π molecular stacking and aggregate sizes) of lignin epoxy resin thermoset materials could be related to the thermomechanical properties via small- and wide-angle X-ray scattering (SAXS and WAXS) [22].

Furthermore, in preparation for material synthesis, it is common in literature for the lignin (technical, depolymerized, and LHO) to undergo chemical modification to improve and tune selective properties by introducing specific chemical functionality. For example, one of the most common methods includes glycidylation which is employed in the preparation of precursors, for example, for lignin epoxy resin materials. For this reason, a second focus of our study was the structural analysis of chemically modified lignin model compounds to gauge how their solid-state structure and the observed intermolecular interactions may change as this would have further implications on modified LHO as a polyol (Figure 1). Lastly, to explore the possible implications of the major interactions observed in all the SCXRD solid-state structures for this study, this would be evaluated with the aid of Coulomb–London–Pauli (CLP) intermolecular energy calculations using the solid-state structures and by measuring their physico-chemical properties using differential scanning calorimetry (DSC) (Figure 1) and comparing these findings to non-crystallizable lignin model compounds.

## 2. Results and Discussion

**Figure 2:**
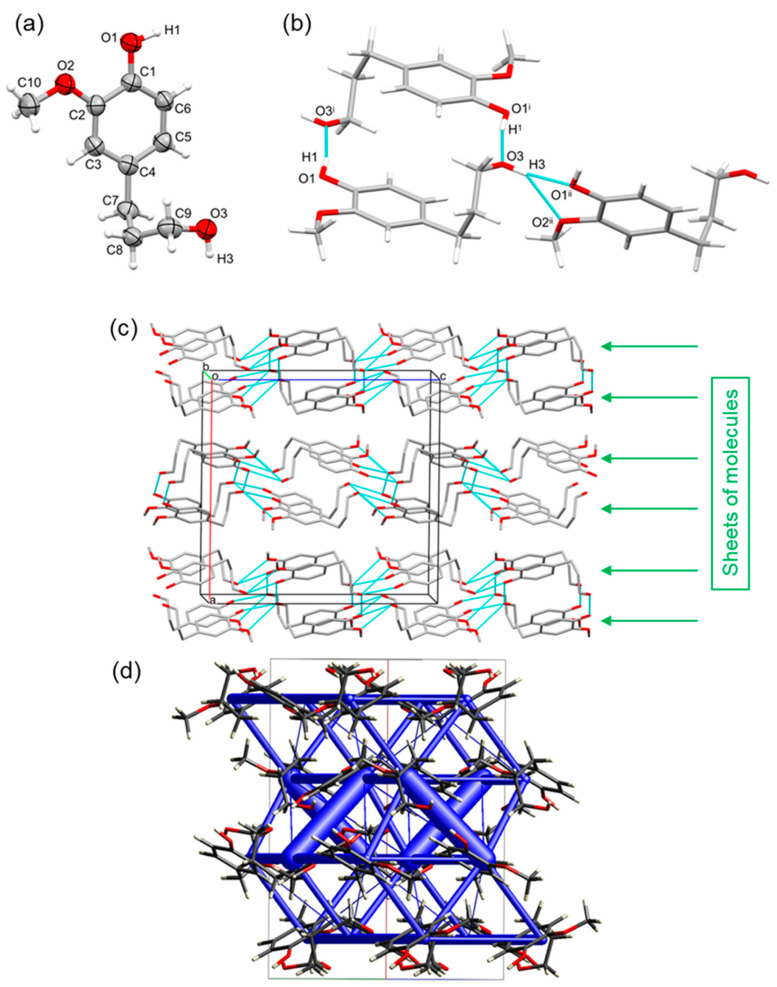
(**a**) Asymmetric unit of dihydroconiferyl alcohol (DCA), showing the labeling scheme of selected atoms and thermal displacement ellipsoids at the 50% probability level. (**b**) H-bond network formed in the packing between phenolic, aliphatic, and methoxy groups of symmetry-equivalent molecules. Twisting of the propyl side-arm allows H-bonding between the aliphatic and aromatic parts. (**c**) Packing in the crystal structure of DCA, showing sheets (highlighted by the green arrows), formed via H-bonding, parallel with the (100) plane. Viewed down the b-axis, H-atoms are omitted for clarity. (**d**) Unit cell of the structure along the bc diagonal showing intermolecular interaction energies as blue tubes. The diameter of the tubes is scaled to the relative stabilization, with the thickest tube being 79.6 kJ/mol.

Recently, we have reported that dihydroconiferyl alcohol (DCA) was a major monomer fraction for the lignin hydrogenolysis oil (LHO) obtained via the RCF of native *Pinus radiata* using a [Pd/C] catalyst (Figure 1) [31,32,35,36]. To better understand the arrangement of this important lignin model, the organic compound was synthesized and recrystallized to obtain the solid-state structure via SCXRD (Figure 2) (see Supplementary Materials [37,38]). DCA crystallized in the orthorhombic centrosymmetric space group *Pbca* (see Supplementary Materials, Table S1 for the data collection and refinement statistics of all solid-state X-ray structures obtained in this study), with one molecule in the asymmetric unit. The two OH alcohol functional groups (chemical functionality of two, ƒ_OH_ = 2), present in the molecule, dictate the arrangement through intermolecular H-bond interactions. Each phenolic OH group interacts with an aliphatic OH group on the propyl side-arm of a symmetry-equivalent molecule [H1∙∙∙O3^i^ = 1.759(19) Å, O1–H1∙∙∙O3^i^ = 168.3(9)°; symmetry code: (i) 1 − x, 1 − y, −z] and vice versa. This dimer has a calculated total stabilization of −79.6 kJ/mol. Each aliphatic OH group interacts with a phenolic OH group and one methoxy O-atom of neighboring molecules [H3∙∙∙O1^ii^ = 2.003(18) Å, O3–H3∙∙∙O1^ii^ = 157.1(11)°, H3∙∙∙O2^ii^ = 2.425(18) Å, O3–H3∙∙∙O2^ii^ = 131.1(11)°; symmetry code: (ii) x, 1/2 − y, −1/2 + z]. This results in the molecules connecting head-to-tail, with a calculated intermolecular energy of −34.7 kJ/mol (Figure 2). Intermolecular π–π stacking interactions (sandwich or T-shape) are minimal and not at play [shortest ring–ring interactions: *Cg*1∙∙∙*Cg*1^i^ = 5.1511(7) Å and *Cg*1∙∙∙*Cg*1^iii^ = 5.2565(7) Å; with *Cg*1 the centroid of the C1–C6 ring; symmetry codes: (i) 1 − x, 1 − y, −z; (iii) 1/2 − x, 1/2 + y, z]. The lack of π–π stacking is to accommodate and maximize the stronger H-bond interactions. Interestingly, this is achieved in the solid-state structure by the propyl side-arms twisting to allow for each aliphatic OH group to align near the planes and vicinity of adjacent aromatic rings and form H-bonds with the two phenolic OH groups (Figure 2). The overall sublimation enthalpy calculated from the solid-state structure through the PIXEL approach with CrystalExplorer (for details, see Supplementary Materials) is 131.5 kJ/mol.

Another prominent monomer compound observed in LHO, in particular, when the RCF catalytic system is shifted from [Pd/C] to [Ru/C], is propyl guaiacol (PG) [6,39,40,41,42]. PG is in the liquid state at room temperature, and, through differential scanning calorimetry (DSC) analysis, a crystallization temperature (*T*_c_) at −32.6 °C and a melting temperature (*T*_m_) at −4.3 °C were observed (see Supplementary Materials for details on the DSC). Unlike for DCA, the trend is reversed for PG, and, upon glycidylation (chemical modification), the propyl guaiacol glycidyl ether (PGGE) (Figure 1) crystallized at room temperature, in agreement with the observations through DSC (*T*_c_ = −14.8 °C and *T*_m_ = 37.3 °C). This was unlike the DCA glycidyl ether (DCAGE) which was a viscous oil at room temperature (*T*_g_ = −39.3 °C). This was unexpected, as commercial epoxides, such as phenyl glycidyl ether (PGE), cyclohexene oxide, propylene oxide, epichlorohydrin, and bisphenol A diglycidyl ether, generally tend to be in the liquid state at room temperature.

**Figure 3:**
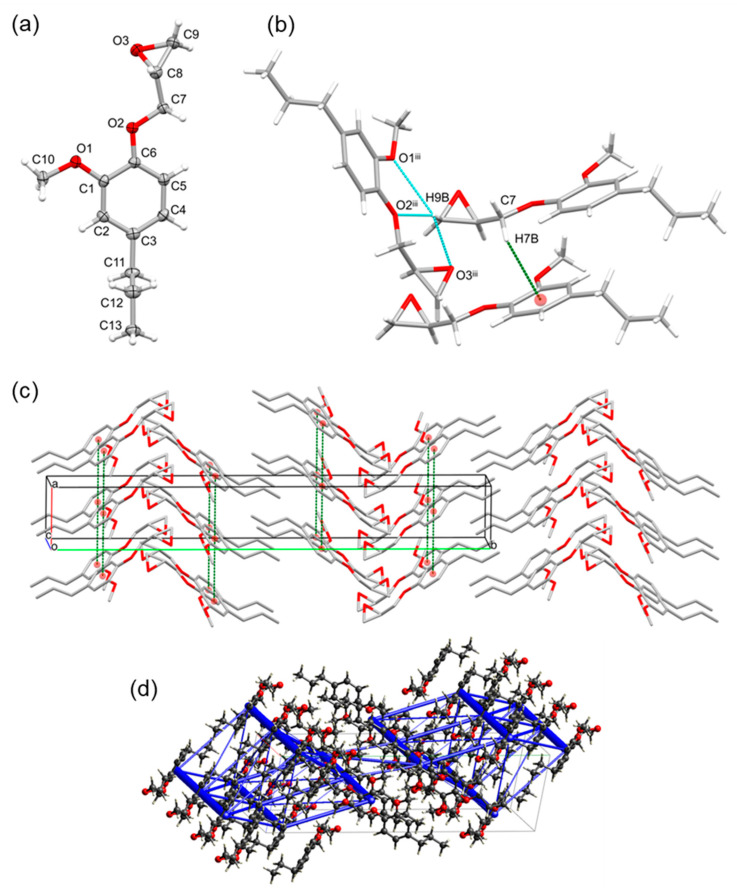
(**a**) Asymmetric unit of propyl guaiacol glycidyl ether (PGGE), showing the labeling scheme of selected atoms and thermal displacement ellipsoids at the 50% probability level. (**b**) Weak H-bond network and C–H∙∙∙π intermolecular interactions observed between symmetry-equivalent molecules. (**c**) Packing in the crystal structure of PGGE, showing stacks of molecules along the [100] direction, via π–π stacking interactions, leading to a typical herringbone pattern. (**d**) Unit cell of the structure showing total intermolecular interaction energies as blue tubes. The diameter of the tubes is scaled to the relative stabilization, with the thickest tube being 50.5 kJ/mol.

PGGE crystallized in the monoclinic centrosymmetric space group *P*2_1_/*n*, with one molecule in the asymmetric unit. As far as we are aware, this is the first single-crystal structure confirmed for a glycidylated lignin model compound that is prominently found in reductively depolymerized lignin oils. In the packing, stacks of molecules are formed along the [100] direction via π–π stacking interactions between one molecule and its upper- and underlying symmetry-equivalent [*Cg*2∙∙∙*Cg*2^i^ = 4.6974(7) Å and *Cg*2∙∙∙*Cg*2^ii^ = 4.6975(7) Å; with Cg2 the centroid of the C1–C6 ring; symmetry codes: (i) −1 + x, y, z; (ii) 1 + x, y, z]. This leads to the strongest intermolecular interactions by far, at −50.5 kJ/mol. The molecular stacks are oriented with their aliphatic and epoxy parts towards each other, leading to a typical herringbone pattern (Figure 3). Furthermore, C–H∙∙∙π intermolecular interactions, like that observed by Vigier et al. for the solid-state structure of the diglycidyl ether of eugenol, are observed between C7–H7B and the phenyl ring [C7–H7B∙∙∙*Cg*2^ii^ = 2.83 Å; with *Cg*2 the centroid of the C1–C6 ring; symmetry code: (ii) 1 + x, y, z] [43]. Despite no OH groups being present here for PGGE, in contrast to DCA, there are weak H-bond interactions between one of the H-atoms of the CH_2_ epoxide group (H9B) and the ether O-atom (O2) of a symmetry-equivalent molecule [H9B∙∙∙O2^iii^ = 2.610 Å, C9–H9B∙∙∙O2^iii^ = 160.12°; symmetry code: (iii) 1/2 + x, 1/2 − y, 1/2 + z]. This leads to the second strongest calculated intermolecular interaction energy of −23.8 kJ/mol, along with interactions between the methoxy groups [H10C∙∙∙O1^i^ = 2.866 Å, C10–H10C∙∙∙O1^i^ = 166.29°; symmetry code: (i) −1 + x, y, z] [44] and the CH entity of the epoxide group with the ether oxygen heteroatom [H8∙∙∙O2^ii^ = 2.749 Å, C8–H8∙∙∙O2^ii^ = 130.04°; symmetry code: (ii) 1 + x, y, z] or methoxy oxygen atom [H8∙∙∙O1^ii^ = 2.829 Å, C8–H8∙∙∙O1^ii^ = 145.90°; symmetry code: (ii) 1 + x, y, z]. However, in these cases, the distances are further apart and the bond angle geometry is less favorable, but comparable to similar H-bond interactions in literature [43,44]. These findings demonstrated clearly how a simple, one-step chemical modification (glycidylation and modification of one phenolic OH group) can have dramatic implications on the structure of the lignin model and, fundamentally, the interactions observed. In addition, these interactions would likely then be present in lignin oils with high concentrations of PG after glycidylation and in a precursor for lignin epoxy resins. Comparing the values for the various components of the total interaction energies, they are clearly dominated by dispersion interactions, in contrast to DCA, where a large part is coulombic, i.e., hydrogen bonding. The total calculated sublimation enthalpy for PGGE is −131.8 kJ/mol.

The eugenol monomer compound is most produced from plant oils, such as clove oil, but can also be produced via the depolymerization or pyrolysis of lignin [9,31,45,46,47,48,49]. Cattey et al. reported the solid-state structure of *iso*-eugenol glycidyl ether but, as far as we are aware, there is no such structure reported for eugenol glycidyl ether (EGE) (Figure 1) [44]. Compound EGE crystallized in the monoclinic centrosymmetric space group *P*2_1_/*n*, with one molecule in the asymmetric unit, and was found to be isomorphous to compound PGGE. A second, triclinic polymorph in the space group *P* − 1 was also found when crystallizing from n-propanol instead of toluene.

**Figure 4:**
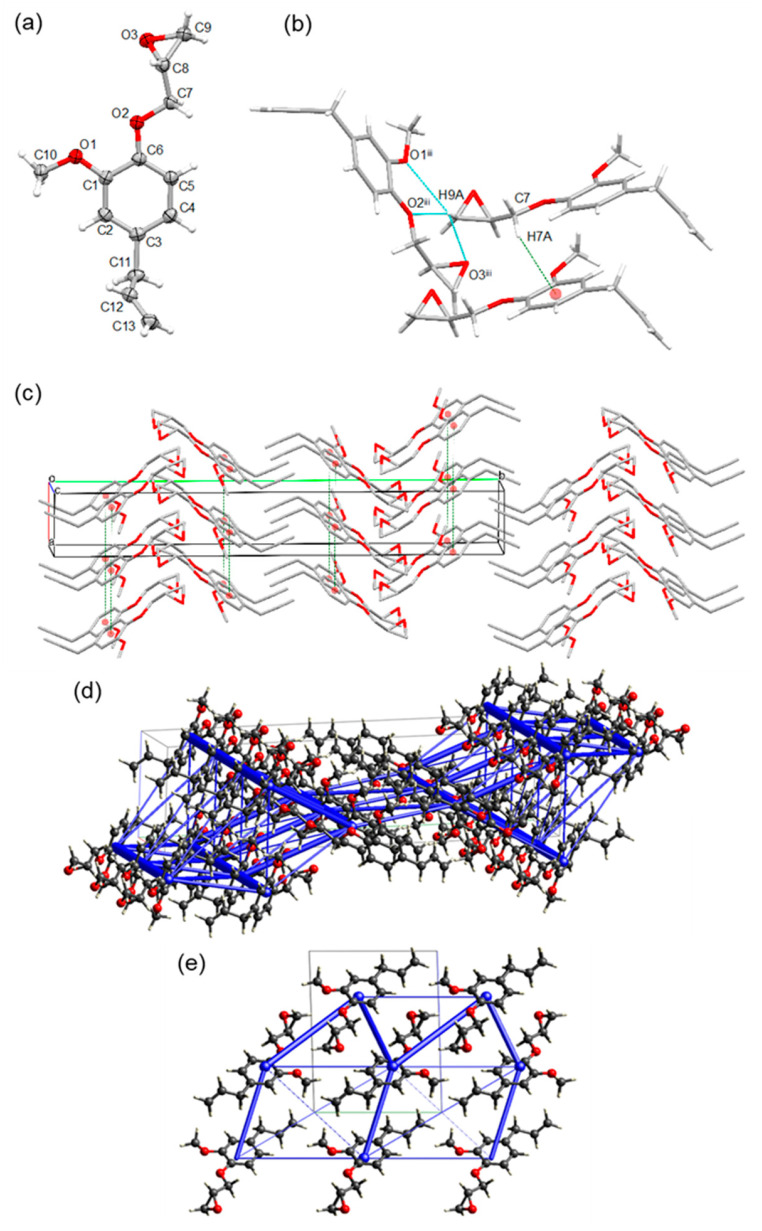
(**a**) Asymmetric unit of eugenol glycidyl ether (EGE), showing the labeling scheme of selected atoms and thermal displacement ellipsoids at the 50% probability level. (**b**) Weak H-bond network and C–H∙∙∙π intermolecular interactions observed between symmetry-equivalent molecules. (**c**) Packing in the crystal structure of EGE, showing stacks of molecules along the [100] direction, via π–π stacking interactions, leading to a typical herringbone pattern. (**d**) Unit cell of the *P*2_1_/*n* EGE structure showing total intermolecular interaction energies as blue tubes. The diameter of the tubes is scaled to the relative stabilization, with the thickest tube being 47.6 kJ/mol. (**e**) Unit cell of the *P* − 1 EGE structure along the a direction, thickest tube 51.9 kJ/mol.

Analogous to PGGE in packing, in the *P*2_1_/*n* structure, stacks of EGE molecules are formed along the [100] direction in the same typical herringbone pattern, via π–π stacking interactions (Figure 4) between one molecule and its upper- and underlying symmetry-equivalent [*Cg*2∙∙∙*Cg*2^i^ = 4.5728(8) Å with *Cg*2 the centroid of the C1–C6 ring; symmetry codes: (i) −1 + x, y, z and 1 + x, y, z]. Furthermore, analogous to PGGE, C–H∙∙∙π intermolecular interactions and weak C–H∙∙∙O interactions are observed (see Figure 4 and Supplementary Materials). The other, *P* − 1, polymorph that crystallizes from polar non-aromatic solvents appears to be less stable, with a calculated sublimation enthalpy of 124.8 kJ/mol vs. 137.3 kJ/mol. The crystal structure is also disordered, with 17% of the end-standing epoxy group being oriented at a 90° angle to its major conformer. The sublimation enthalpy was calculated for 100% occupancy of the major conformer. In this case, the most stabilizing interactions are, again, π–π stacking, [*Cg*3∙∙∙*Cg*3^i^ = 4.800(2) Å with *Cg*3 the centroid of the C1–C6 ring; symmetry code (i) −1 + x, y, z], for an interaction energy of −51.9 kJ/mol, followed by a set of interactions between an epoxy CH and a methoxy O-atom, over the inversion center (symmetry code −x, −y, −z), for a total of −31.6 kJ/mol. Moreover, here, there are additional CH–π and CH–O contacts. If the crystal is completely populated with minor conformers, it is clear there is a repulsive contact between the epoxides, to the tune of +27.1 kJ/mol. The minimum energy for the crystal (lower than with only major conformer present) will be obtained if the structure can be relaxed with the 17% observed disorder, but this is hard to demonstrate computationally, as it destroys the long-range order in the crystal and requires many more molecules as a supercell.

The only difference, chemically, between the PGGE and EGE molecules is the C3 side chains, with EGE containing a terminal alkenyl group. The difference in the hybridization for C12 and C13 atoms does have subtle implications on the bond and torsion angles, and, as a result, the stability of the crystal. For PGGE, the geometry of the tetrahedral *sp*^3^ atoms enables more flexibility and the C12–C13 bond aligns parallelly to the plane of the benzene ring with the propyl arm rotating perpendicular from the aromaticity [C3–C11–C12–C13 = −178.31(12)°]. This minimizes steric repulsion, within the molecule and with the adjacent molecule in the unit cell, where the C12–C13 bond is pointing away and is parallel with the adjacent benzene ring of the other pairing neighbor molecule (Figure S1). This allows for an energetically more stable packing of the crystal. For EGE, the trigonal planar *sp*^2^ C12 and C13 atoms and C12–C13 are is more constrained by the increased electron density and cannot rotate as effectively away from the benzene rings and planes [C3–C11–C12–C13 = 128.01(15)° for the *P*2_1_/*n* structure, and C4–C7–C8–C9 = 129.9(4)° for the *P* − 1 structure] (Figure S1). This results in the propyl side-arms pointing toward the benzene ring of adjacent molecules, greater steric repulsion, and a less energetically stable packing of the crystal (Figure S1 highlighted in red). In addition, it seems that the double bond cannot partake in any stabilizing π interactions, such as with the benzene ring of the adjacent molecule, because the propenyl chain is not long enough.

These crystallographic observations for PGGE and EGE are consistent with the visible observations and physical properties measured via DSC. The PGGE crystals were more stable at room temperature and easier to mount on the diffractometer compared to EGE. While attempting to recrystallize the EGE, any crystals formed were less stable and prone to melting if the room temperature was increased. Thus, they were more challenging to run for X-ray diffraction; having to be stored in the fridge and placed on ice before mounting. DSC analysis corroborated these observations with a lower *T*_m_ measured of 32.6 °C and a higher *T*_c_ of −5.7 °C for EGE compared to PGGE (*T*_c_ = −14.8 °C and *T*_m_ = 37.3 °C). The DSC trace for EGE shows a second melting peak, conceivably for the *P* − 1 structure around 28 °C. The higher melting point for the PGGE crystals is indicative of a more energetically stable and more closely packed crystal. The calculated PIXEL lattice energies on the solid-state structures seem to suggest the opposite trend, with −131.8 kJ/mol for PGGE, and 137.25 and −124.75 kJ/mol for the *P*2_1_/*n* and *P* − 1 polymorphs for EGE, respectively. It needs to be kept in mind that these energies correspond to *sublimation* enthalpies, that do not take interactions occurring in the liquid phase into account. The difference in melting point(s) can be explained if we assume fewer or less strong interactions in the liquid phase for PGGE, which makes sense, as its propyl side chain is limited to dispersion interactions. This was an interesting example of how crystallography could be related to and used to explain the physical properties of a chemical—specifically, in this instance, chemically modified lignin model compounds that would be found in chemically modified LHO. This also demonstrated how a subtle difference in a chemical structure, such as alkyl vs. alkenyl end-group functionality, results in distinct differences in the stability of the crystal and the chemical’s physical properties.

Furthermore, it was unexpected that PGGE and EGE should crystallize when propyl guaiacol (PG) and eugenol (E), with aliphatic OH groups present for H∙∙∙O–H bonding interactions, exist in the liquid state at room temperature. It can only be hypothesized in this instance that the glycidylation of propyl guaiacol and eugenol, and the modification of the phenolic OH group on the molecule, increases the overall attractive intermolecular forces to form a well-packed solid crystal; the combination of H-bonding from the ether oxygen heteroatom, epoxide, and methoxy groups for PGGE and EGE were stronger than that of the H-bonding from the aliphatic OH group for PG and E. On the other hand, this trend is reversed for DCA. At room temperature, DCA exists as a crystalline solid and the large number of H-bonding interactions stabilize both the solid and crystal (Figure 2). However, if the DCA is chemically modified and the phenolic OH groups are epoxidized, resulting in only one (aliphatic) OH group being present in the molecule (ƒ_OH_ = 1), the DCA glycidyl ether (DCAGE) would be a viscous oil at room temperature which would not solidify and attempts at recrystallisation would be unsuccessful. Through DSC, a *T*_g_ of −39.3 °C was observed between −60 to 70 °C. It was postulated that the removal of half of the OH groups and H-bonding interactions destabilized the network and resulted in an insufficient amount of attractive intermolecular forces to keep the solid together, resulting in the observed viscous liquid. This is an important finding as it will have implications on the properties of the LHO, such as the viscosity, which will be dependent on the proportion of monomers that were present in the LHO (for example, DCA or PG). In addition, these properties will again dramatically change upon the glycidylation of the LHO; all of these points need consideration for material synthesis and applications for the LHO.

**Figure 5:**
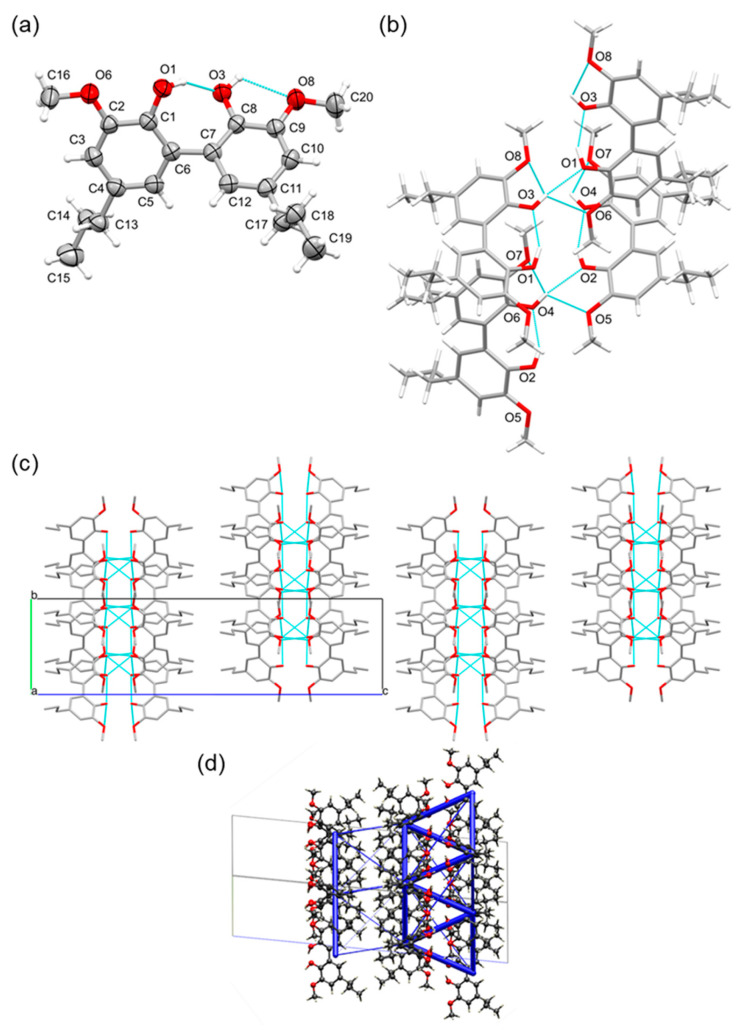
(**a**) Molecular structure of the propyl guaiacol dimer (PGD), showing the labeling scheme of selected atoms and thermal displacement ellipsoids at the 50% probability level (only one molecule of the asymmetric unit is shown). (**b**) Extensive intra- and intermolecular H-bond network observed between symmetry-equivalent molecules. (**c**) Packing in the crystal structure of PGD, showing double-layered molecular sheets, parallel with the (001) plane, formed by extensive intermolecular H-bond interactions along the [100] and [010] directions; H-atoms are omitted for clarity. (**d**) Structure along the ab diagonal of the unit cell, showing total intermolecular interaction energies as blue tubes. The diameter of the tubes is scaled to the relative stabilization, with the thickest tube being 48.4 kJ/mol.

Of the compounds observed for the dimer fraction of LHO, obtained via RCF, the C–C condensed, 5–5′ propyl guaiacol dimer (PGD) (Figure 1) is often identified in reports [31,41,50,51,52]. Upon the synthesis of the PGD (see Supplementary Materials), the model bore a resemblance to DCA, being a crystalline solid at room temperature and when epoxidized; when removing the phenolic OH groups, it existed as a viscous oil at room temperature. These observations agreed with those detected via DSC (PGD; *T*_c_ = 126.2 °C, *T*_m_ = 147.1 °C vs. PGD diepoxide; *T*_g_ = −16.4 °C). Thus, it was possible to obtain the solid-state structure of the PG dimer (PGD) (Figure 1), and not of the epoxidized dimer. PGD crystallized in the monoclinic centrosymmetric space group *P*2_1_/*n* and the asymmetric unit contains two complete dimer molecules. As was the case with DCA, the solid-state structure of the PGD shows extensive H-bonding interactions, along the [100] and [010] directions, forming double-layered molecular sheets, parallel with the (001) plane (Figure 5).

In fact, intramolecular H-bonds are formed between hydroxyl groups [H1∙∙∙O3 = 1.89(3) Å, O1–H1∙∙∙O3 = 151.2(16)°; H2∙∙∙O4 = 1.94(3) Å, O2–H2∙∙∙O4 = 151.4(16)°] and between hydroxyl and methoxy groups [H3∙∙∙O8 = 2.21(2) Å, O3–H3∙∙∙O8 = 114.0(17)°, H4∙∙∙O7 = 2.20(2) Å, O4–H4∙∙∙O7 = 113.7(16)°] (Figure 5). Intermolecular H-bonds are also formed between hydroxyl groups [H3∙∙∙O1^i^ = 2.18(2) Å, O3–H3∙∙∙O1^i^ = 154.1(19)°, H4∙∙∙O2^ii^ = 2.15(2) Å, O4–H4∙∙∙O2^ii^ = 154.7(17); symmetry codes: (i) 1/2 − x, 1/2 + y, 3/2 − z, (ii) 3/2 − x, 1/2 + y, 3/2 − z] and between hydroxyl and methoxy groups [H3∙∙∙O6^i^ = 2.48(2) Å, O3–H3∙∙∙O6^i^ = 119(2)°, H4∙∙∙O5^ii^ = 2.48(2) Å, O4–H4∙∙∙O5^ii^ = 116(2)°; symmetry codes: (i) 1/2 − x, 1/2 + y, 3/2 − z, (ii) 3/2 − x, 1/2 + y, 3/2 − z] (Figure 5). The strongest, shortest H-bonds were the intramolecular interactions between hydroxyl groups (H1∙∙∙O3 and H2∙∙∙O4) because a seven-member cyclic ring is partially formed and stabilized via the chelate effect.

This large number of intermolecular contacts entails a dramatic effect on the packing of the crystal structure. To maximize these H-bonding interactions (particularly between the phenolic groups of the same molecule), the network grows along the [100] and [010] directions via the arrangement of the dimer molecules in space and torsion of the benzene rings along the C6–C7 and C21–C27 dimer bonds [torsion angles C1–C6–C7–C8 = −51.0(3)° and C22–C21–C27–C28 = −50.8(3)°] to prevent any relevant π–π stacking interactions (centroid–centroid distances between benzene rings are in the range of 5.6157(11)–5.6496(11) Å). To minimize the steric repulsion between molecules, the two aliphatic propyl arms of each molecule are directed towards the other aliphatic arms of symmetry-equivalent molecules in the packing. Furthermore, weak C–H∙∙∙π intermolecular interactions are observed between the four methoxy CH3 hydrogen atoms and the four benzene rings [C(–H)∙∙∙*Cg* distances in the range of 3.583(3)–3.590(3) Å]. The lattice energy for both symmetry-independent molecules is −191.45 and −190.05 kJ/mol, so very similar. As expected from the geometry, interactions between, as well as within, the H-bonded layers are substantial, whereas the double layers flanked with propyl groups on either side have very little contribution to the lattice energy.

**Figure 6:**
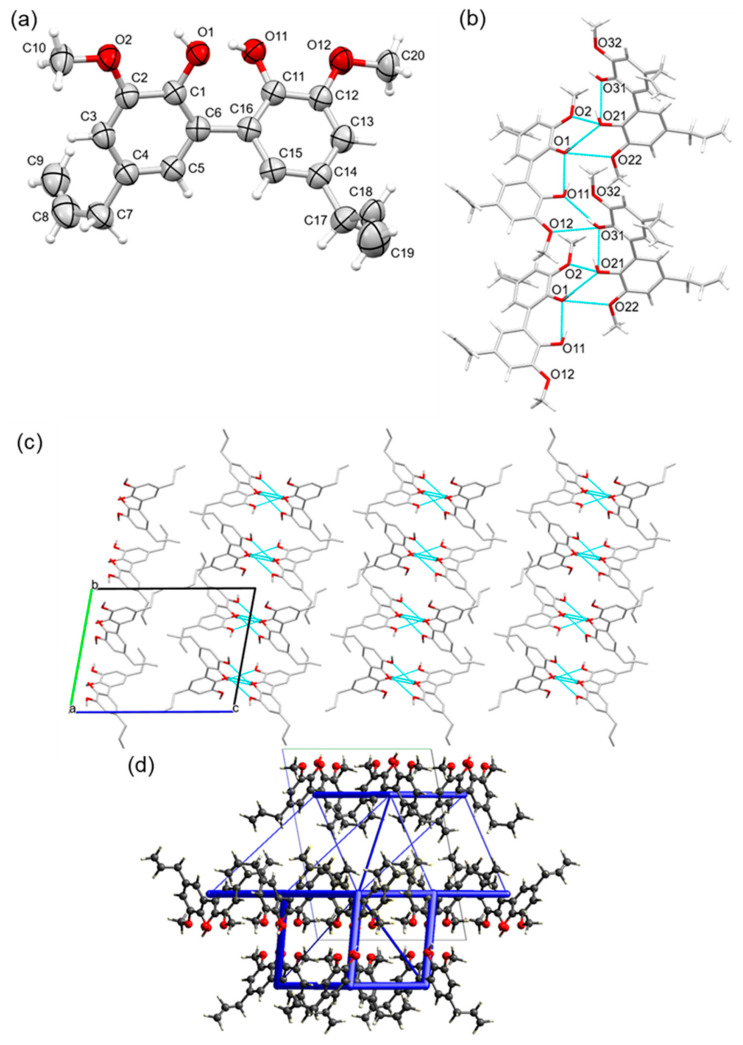
(**a**) Molecular structure of the eugenol dimer (ED), showing the labeling scheme of selected atoms and thermal displacement ellipsoids at the 30% probability level (only one molecule of the asymmetric unit is shown). Disorder of the propylene side-arm parts is omitted for clarity. (**b**) Extensive intra- and intermolecular H-bond network observed between symmetry-equivalent molecules. (**c**) Packing in the crystal structure of ED, showing double-layered molecular sheets, parallel with the (001) plane, formed by extensive intermolecular H-bond interactions along the [100] and [010] directions; H-atoms are omitted for clarity. (**d**) Total intermolecular energy frameworks in the structure, shown along the a axis. Tubes are scaled according to the strength of the interactions. Strongest interactions are 47.6 kJ/mol between molecules in the H-bonded layers.

The eugenol dimer (ED, Figure 1) solid-state structure was also obtained (Figure 6). However, unlike the isomorphous PGGE and EGE structures, ED crystallized in the triclinic centrosymmetric space group *P* − 1 and the asymmetric unit contains two dimer molecules. Despite these differences, the H-bond interactions were similar to the PGD, forming along the [100] and [010] directions with double-layered molecular sheets observed parallel with the (001) plane. However, in this case, there were no noticeable C–H∙∙∙π interactions occurring.

For the ED, both the *T*_c_ and *T*_m_ values were observed to be distinctly lower in temperature for the DSC analysis compared to the PGD, suggesting that both the ED solid-state structure was less stable and the attractive forces were weaker in comparison (ED; *T*_c_ = 67.9 °C, *T*_m_ = 96.8 °C vs. PGD; *T*_c_ = 126.2 °C, *T*_m_ = 147.1 °C). This was attributed to the less flexible propyl side-arms for ED resulting in more steric repulsion, in comparison to PGD, when the aliphatic propyl arms of each molecule are directed towards the other aliphatic arms of other molecules in the packing. In the CLP energy calculations (see Supplementary Materials for details) of the solid-state structure, both ED molecules show disorder in the propylene side chain. Only the crystal composed of major conformers has been calculated, and results in a stabilizing energy of −174.05 kJ/mol for one of the molecules in the asymmetric unit, and −171.6 kJ/mol for the other. These numbers may be on the less stabilized side because the presence of the disorder in the propylene chain suggests that, in reality, a deeper energy minimum is possible. Nevertheless, the difference with PGD (~190 kJ/mol) is in accordance with the observed difference in melting points of 50 °C between ED and PGD.

**Figure 7:**
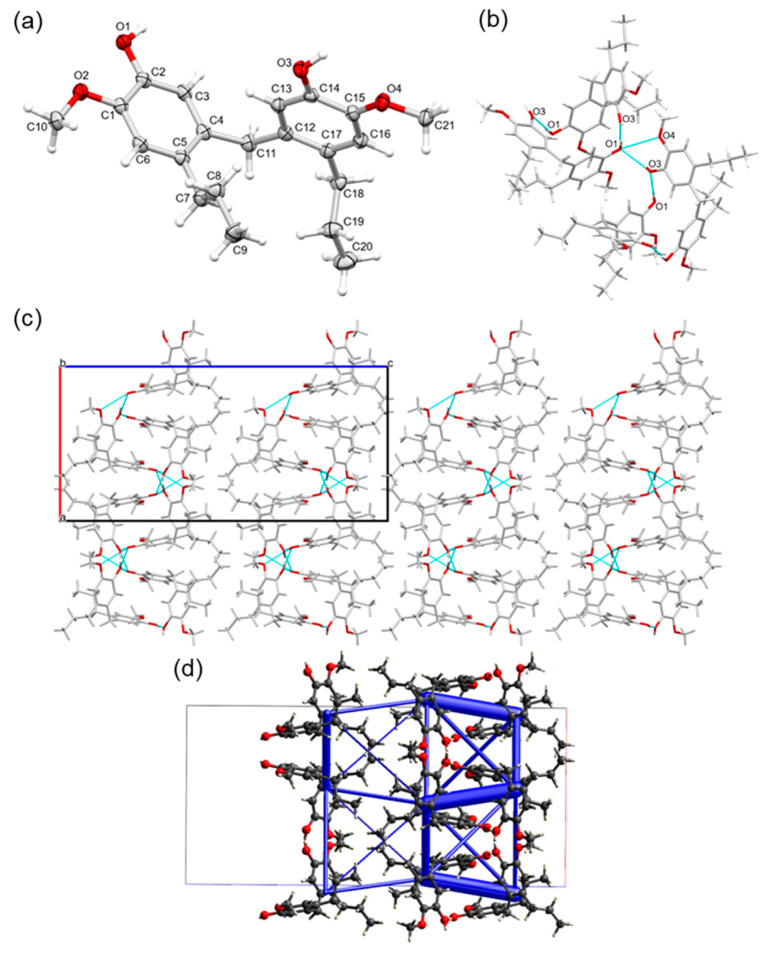
(**a**) Molecular structure of the methylene propyl guaiacol dimer (MPGD), showing the labeling scheme of selected atoms and thermal displacement ellipsoids at the 50% probability level. (**b**) Extensive intra- and intermolecular H-bond network observed between symmetry-equivalent molecules. (**c**) Packing in the crystal structure of MPGD, showing double-layered molecular sheets, parallel with the (001) plane, formed by extensive intermolecular H-bond interactions along the [100] and [010] directions. (**d**) Energy frameworks in the MPGD structure, shown along the b axis. Tubes are scaled according to the strength of the interactions. Strongest interactions are 80.0 kJ/mol.

In addition to the glycidylation previously discussed, another important chemical modification of lignin is its reaction with formaldehyde in the preparation of precursors, for example, phenol-formaldehyde (PF) or phenolic thermosetting resins [5,34,53,54,55,56,57,58]. Phenolic resins are used for a number of industrial applications such as in the production of plywood, cardboard, and composite materials [34,55,56,57,58]. Therefore, for further study, the methylene propyl guaiacol dimer (MPGD, Figure 1) was synthesized from propyl guaiacol and the solid-state structure was obtained (Figure 7). Compound MPGD crystallized in the orthorhombic centrosymmetric space group *Pbcn* with one molecule in the asymmetric unit. This methylene dimer bears a resemblance to PGD and ED with extensive H-bonding interactions, along the [100] and [010] directions, forming double-layered molecular sheets, parallel with the (001) plane (Figure 7).

As was the case for the PGD, intermolecular bonds are formed between hydroxyl groups for MPGD [H1∙∙∙O3^i^ = 1.914(17) Å, O1–H1∙∙∙O3^i^ = 172.0(14)°, H3∙∙∙O1^ii^ = 1.909(15) Å, O3–H3∙∙∙O1^ii^ = 164.3(15)°; symmetry codes: (i) 1 − x, y, 3/2 − z, (ii) 1/2 + x, 1/2 + y, 3/2 − z] (Figure 7). However, the methylene moiety in between the aromatic PG–PG bridge has a considerable influence on the packing, with no intramolecular H-bonds formed because of the increased separation between the hydroxy groups of the same molecule [O(1)∙∙∙O(3) = 6.723(1) Å]. As a result of the absence of this intramolecular H-bond, there is no conformational lock between the phenolic groups of the same molecule, and the benzene rings rotate almost perpendicular relative to each other to maximize the intermolecular H-bond interactions [C(3)–C(4)–C(11)–C(12) = 100.11(12)°]. As another consequence, adjacent benzene rings are offset relative to one another with no clear intermolecular π–π stacking interactions observed [shortest ring–ring interactions: *Cg*1∙∙∙*Cg*1^i^ = 4.7774(6) Å, *Cg*1∙∙∙*Cg*1^iii^ = 5.5264(6) Å and *Cg*1∙∙∙*Cg*2^iv^ = 4.8733(7) Å; with *Cg*1 the centroid of the C1–C6 ring and *Cg*2 the centroid of the C12–C17 ring; dihedral angle between the two planes: *Cg*1∙∙∙*Cg*1^i^ = 20.62(5)°, *Cg*1∙∙∙*Cg*1^iii^ = 29.97(5)° and *Cg*1∙∙∙*Cg*2^iv^ = 85.05(5)°; symmetry codes: (i) 1 − x, y, 3/2 − z, (iii) 1/2 − x, −1/2 + y, z, (iv) x, y, z] and no C–H∙∙∙π interactions observed.

Unlike PGD, interactions from the methoxy groups were less prevalent in the crystal packing for MPGD. There were no intermolecular O–H∙∙∙O hydrogen bonds observed from this group but there was a intramolecular H-bond formed between a hydroxyl group and methoxy group [H3∙∙∙O4 = 2.359(15) Å, O3–H3∙∙∙O4 = 106.1(12)°] (Figure 7). The other methoxy group (O2) is not involved in any noticeable interactions.

A summary of the main findings and observations made by this study, via SCXRD and/or DSC, are shown in Table 1, a demonstration of how crystallography can be used to help relate the solid-state structure and molecular arrangement of chemical compounds to their physical properties, in this case, focusing on lignin model compounds that are major monomer and dimer compounds commonly present in depolymerized LHOs, or after chemical modifications commonly applied to lignin (glycidylation or formaldehyde). The DCA monomer was a stable solid at room temperature because of the extensive H-bonding network present, as observed visibly and via DSC (*T*_m_ = 61.7 °C). However, after the first heating signal and melting, upon the second heating cycle, no *T*_m_ was observed but a *T*_g_ at −32.5 °C. There were no changes in the thermogram upon slowing the cooling rate. As there was no solvent of crystallization observed in the SCXRD that would evaporate during the first heating cycle, this was hypothesized to result from the hydrogen bonds being broken and not being able to reform before the second heating cycle, with the sample existing or remaining as a viscous oil. This differs from the PG monomer which existed in the liquid state at room temperature. It was assumed that the only major intermolecular forces present in the molecules would be H-bonding and, thus, with only one OH group present in the molecule, compared to DCA (PG, ƒ_OH_ = 1 vs. DCA, ƒ_OH_ = 2), the weaker attractive forces were unable to bring the molecules together and form a solid at room temperature. Indeed, crystallization was observed to be only possible at cooler temperatures (*T*_c_ = −32.6 °C) but the crystal lattice was unstable, weak, and dissociated quickly upon warming (*T*_m_ = −4.3 °C). Nothing was observed using DSC for the E liquid monomer. This possibly implied the attractive forces were weaker here compared to PG, and, hence, any possible crystal would be less stable than that of PG with both *T*_c_ and *T*_m_ occurring before −60 °C.

The lignin dimer compounds, DCA dimer, PGD, and ED, were stable solids and had *T*_c_/*T*_m_ values well above room temperature (Table 1). Interestingly the PGD seemed more stable than the DCA dimer, via observations through DSC, despite the lower number of OH groups present (PGD, ƒ_OH_ = 2, *T*_c_ = 126.2 °C, *T*_m_ = 147.1 °C vs. DCA dimer, ƒ_OH_ = 4, *T*_c_ = 96.0 °C, *T*_m_ = 135.5 °C). It would have been perceived that this would decrease the attractive forces present because of less H∙∙∙O–H bonding, and decrease the *T*_c_/*T*_m_ values. However, there must be other additional attractive forces at play in the molecular organization of the PGD solid to result in the higher *T*_c_ and *T*_m_, as also evidenced from the lattice energy calculations. From the analysis of the PGGE and EGE solid-state structures, this may possibly result from C–H∙∙∙O bonding and/or C–H∙∙∙ π interactions. In contrast to the other dimer molecules, MPGD followed a similar trend to the DCA monomer with a *T*_m_ measured at 81.0 °C for the first heating cycle, and, upon melting and the second heating cycle, no *T*_m_ was observed but a *T*_g_ at −4.1 °C. The lower value and disappearance of the *T*_m_ indicated a less stable H-bonding network present that could not reform rapidly once broken with the methylene bridge, causing more instability for the MPGD in comparison to PGD and ED. This is an important consideration as it demonstrates just how the physical properties and molecular organization will distinctly change if LHO is chemically modified for resin preparation.

As mentioned earlier, the most unexpected finding of this study was that, when PG and E were chemically modified via glycidylation (PGGE and EGE), the products were solids at cooler room temperatures and could be recrystallized. This is despite commonly used commercial epoxides typically existing in the liquid state (for example, cyclohexene oxide, propylene oxide, styrene oxide, epichlorohydrin, allyl glycidyl ether, and phenyl glycidyl ether). Furthermore, DCAGE consisting of one OH aliphatic group capable of H-bonding (O–H∙∙∙H) was a viscous oil at room temperature and no *T*_c_ or *T*_m_ was observed (Table 1). As discussed earlier, this finding for PGGE and EGE was attributed to attractive C–H∙∙∙O bonding and C–H∙∙∙ π interactions. EGE was less stable as a solid, both visibly and via DSC, with a smaller *T*_m_ and difference between the *T*_c_ and *T*_m_ values [Δ(*T*_m_ − *T*_c_); PGGE = 52.1 °C vs. EGE = 38.3 °C]. On the other hand, when the PGD was epoxidized, removing the two phenolic OH groups and O–H∙∙∙H bonding, a viscous oil was produced at room temperature (*T*_g_ = −16.4 °C and no *T*_c_ or *T*_m_). The attractive forces present for PGGE and EGE could not occur for the PGD diepoxide, possibly because of the larger dimer molecules being too sterically bulky and less mobile to organize. Commercial PGE was also analyzed via DSC (*T*_c_ = −25.4 °C, *T*_m_ = −9.7 and −4.8 °C) and was consistent with being a liquid at room temperature. The small difference in *T*_c_ and *T*_m_ highlights PGE’s instability when crystallized [Δ(*T*_m_ − *T*_c_) = 15.7 °C]. Excluding the alkyl chain, a major difference between PGE (liquid) and PGGE (solid) is the lack of a methoxide group present and, therefore, the lower possibility for C–H∙∙∙O and/or C–H∙∙∙ π interactions. This is a noteworthy mention because, as well as for X-ray crystallography, methoxide groups can have dramatic effects on material properties. Hernandez et al. observed that changing the structure of a vanillyl-alcohol-derived diglycidyl ether bisguaiacol epoxide influenced the thermomechanical properties of the corresponding epoxy resin material; the introduction of methoxy groups would lower the *T*_g_ and increase the glassy storage modulus at 25 °C [59]. This decrease in the *T*_g_ value, because of the presence of methoxy groups, is consistent with that reported for lignin-derived epoxy resin materials [31,32,36,60,61].

## 3. Conclusions

Single-crystal X-ray diffraction was applied to the synthesized lignin model compounds, dihydroconiferyl alcohol and, propyl guaiacol, and eugenol dimers, that are commonly found in lignin hydrogenolysis oil. As was expected, intermolecular H-bonding interactions dictated the observed solid-state structures with a molecular arrangement prioritizing these fundamental interactions. The solid-state structures were also confirmed for the chemically modified lignin models, methylene bridging propyl guaiacol dimer, propyl guaiacol glycidyl ether, and eugenol glycidyl ether; these species would be likely present in lignin precursors that can be used for epoxy and phenol-formaldehyde resins and material applications. Despite the removal of OH groups, the epoxides unexpectedly crystallized via the observed π–π stacking, C–H∙∙∙π, and weaker C–H∙∙∙O bonding interactions. This was important to observe because OH groups would be removed when lignin oil is applied as a polyol for material applications with these π interactions possibly being present.

This is also of interest because, although H-bonding was dominant in the solid-state structures for unmodified monomers and dimers and this is likely to be the case in depolymerized lignin oils, in both technical and native lignin, the structure would be, of course, distinctly different. With no depolymerization and breaking of C–O ether bonds, there would be substantially fewer OH groups, with these π interactions being more prevalent. It is common knowledge that the different types of lignin (technical vs. biorefinery (lignin-first)) create very different materials with different properties. This is often attributed to differences in factors such as molecular weight, solubility, or chemical functionality. However, these aspects are all fundamentally related to the molecular organization and intermolecular interactions, which is commonly understated in literature. For example, for lignin hydrogenolysis oils, a decrease in the molecular weight implies an increase in the [monomer][dimer]/[oligomer] ratio, for the compounds in the mixture, and likely the number of H-bonding interactions. This would result in the solubility increasing in organic solvents and influencing the thermomechanical properties of the resulting materials synthesized from the LHOs.

During the study, there were unexpected trends for which lignin model chemicals would recrystallize. This was further explored with the aid of DSC analysis and CLP intermolecular energy calculations, where the relationship between the chemical structure, functionality (ƒ_OH_), the major interactions observed in the SCXRD solid-state structures, and the physico-chemical properties (*T*_m_, *T*_c_ and *T*_g_) were evaluated for a range of lignin model compounds. These considerations could have several implications for the lignin community. For instance, in view of improving the lignin biorefining processes and the valorization of lignin, improving our knowledge on the crystallization profile of model compounds and better understanding the subsequent interactions that would likely be present in the molecular organization of the solid-state structures for more complex lignin oils as such (beyond model compounds) could improve downstream processing applications, such as in enhancing the separation and isolation of specific bioaromatic compounds and/or for the future fine-tuning of the thermo-mechanical properties of lignin-based materials, for industrial material applications, by the more efficient design and better understanding of lignin oil polyols. Currently, these fundamental interactions are hidden from us because of the amorphous nature of actual lignin (native, technical, and depolymerized), preventing the direct application of SCXRD beyond model compounds, and this presents a knowledge gap that needs further insight.

## Figures and Tables

**Table 1:** Summary of the lignin model compounds analyzed via SCXRD, CLP-calculated sublimation enthalpy (with electron density at B3LYP/6-31(d,p) level), and/or DSC analysis.

Lignin Model	Lignin Compound	Physical State at 25 °C	ƒ_OH_	Major Attractive Interactions Observed via Single-Crystal X-ray Diffraction	Physical Properties Observed via DSC	CLP-Calculated Sublimation Enthalpy	Total Energy/ kJ/g
Monomer	DCA	Solid	2	H-bonding (O–H∙∙∙O)	*T*_m_ = 61.7 °C ^a^, *T*_g_ = −32.5 °C ^b^	−131.5 kJ/mol	−0.722
	PG	Liquid	1	n.d. (Assume H-bonding occurring)	*T*_c_ = −32.6 °C, *T*_m_ = −4.3 °C	-	-
	E	Liquid	1	n.d. (Assume H-bonding occurring)	Nothing observed	-	-
Dimer	DCA dimer	Solid	4	n.d. (Assume H-bonding occurring)	*T*_c_ = 96.0 °C, *T*_m_ = 135.5 °C	-	-
	PGD	Solid	2	H-bonding (O–H∙∙∙O) and π interactions (C–H∙∙∙π)	*T*_c_ = 126.2 °C, *T*_m_ = 147.1 °C	−190.1 kJ/mol and −191.5 kJ/mol	−0.577
	ED	Solid	2	H-bonding (O–H∙∙∙O)	*T*_c_ = 67.9 °C, *T*_m_ = 96.8 °C	−174.1 kJ/mol and −171.6 kJ/mol	−0.533
Chemically modified	DCAGE	Viscous liquid	1	n.d. (Attempts at recrystallization were unsuccessful)	*T*_g_ = −39.3 °C	-	-
	PGGE	Solid	0	H-bonding (C–H∙∙∙O) and π interactions (π–π, C–H∙∙∙ π)	*T*_c_ = −14.8 °C, *T*_m_ = 37.3 °C	−131.8 kJ/mol	−0.593
	EGE	Solid	0	H-bonding (C–H∙∙∙O) and π interactions (π–π, C–H∙∙∙ π)	*T*_c_ = −5.7 °C, *T*_m_ = 28 °C, 37.3 °C	Monoclinic: −137.3 kJ/mol Triclinic: −124.8 kJ/mol	−0.623 −0.566
	PGD diepoxide	Viscous liquid	0	n.d.	*T*_g_ = −16.4 °C	-	-
	MPGD	Solid	2	H-bonding (O–H∙∙∙O)	*T*_m_ = 81.0 °C ^a^, *T*_g_ = −4.1 °C ^b^	−186.0 kJ/mol	−0.519
Commercial	PGE	Liquid	0	n.d.	*T*_c_ = −25.4 °C, *T*_m_ = −9.7 and −4.8 °C	-	-

Unless stated otherwise, *T*_g_, *T*_c_, and *T*_m_ values were determined from the 2nd heating cycle between −60 °C to 70 °C or 200 °C. ^a^ Observed in the 1st heating cycle. ^b^ Observed in the 2nd heating cycle. Abbreviations: n.d. = not determined or could not be determined. ƒ_OH_ = chemical functionality or number of OH groups.

## Data Availability

The data will be made available upon request. The experimental section, methods, materials, and additional data concerning the synthesis and characterization of the lignin model compounds, DSC (Figures S9–S20), FT–IR (Figures S2–S8), CLP energy calculations at the B3LYP/6-31(d,p) electron density level, and additional crystallographic tables and data can be found in the supporting information. The SCXRD solid-state structures and supplementary crystallographic experimental data (CCDC numbers 2359588–2359590 and 2361560–2361563) can be obtained as a .cif file from The Cambridge Crystallographic Data Centre via www.ccdc.cam.ac.uk/structures (accessed on 26 September 2024).

## Supplementary Materials

The supporting information can be downloaded at: https://www.mdpi.com/article/10.3390/ijms251910810/s1.
