# Supplementary material for: Solid-State Structures and Properties of Lignin Hydrogenolysis Oil Compounds: Shedding a Unique Light on Lignin Valorization
Source: Int J Mol Sci. 2024 Oct 8;25(19):10810. doi: 10.3390/ijms251910810 (PMC11477037; doi:10.3390/ijms251910810)
Supplement: Supplementary file 1 [file ijms-25-10810-s001.zip › ijms-3221285-supplementary.pdf]

Supplementary Information for

Solid-State Structures and Properties of Lignin Hydrogenolysis Oil Compounds: Shedding a Unique Light on Lignin Valorization

Oliver J. Driscoll [1,2,*], Kristof Van Hecke [3], Christophe M. L. Vande Velde [4], Frank Blockhuys [5], Maarten Rubens [1], Tatsuhiro Kuwaba [1], Daniel J. van de Pas [2], Walter Eevers [1,5], Richard Vendamme [1] and Elias Feghali [1,6,*]

1 Sustainable Polymer Technologies Team, Flemish Institute for Technological Research (Vito N.V.), Boeretang 200, 2400 Mol, Belgium

2 New Zealand Forest Research Institute (Scion), Private Bag 3020, Rotorua 3046, New Zealand

3 XStruct, Department of Chemistry, Ghent University, Krijgslaan 281-S3, 9000 Ghent, Belgium

4 iPRACS, Faculty of Applied Engineering, University of Antwerp, Groenenborgerlaan 171, 2020 Antwerp, Belgium

5 Department of Chemistry, University of Antwerp, Groenenborgerlaan 171, 2020 Antwerp, Belgium

6 Chemical Engineering Program, Notre Dame University–Louaize, Zouk Mosbeh 1211, Lebanon

* Correspondence: oli.driscoll@vito.be (O.J.D.); elias.feghali@vito.be (E.F.)

Contents

Comparison between PGGE and EGE 3

Materials and methods 3

Lignin Model Compound Synthesis and Characterization 7

IR Spectra of Lignin Model Compounds 12

DSC Curves of Lignin Model Compounds 16

Crystallographic Tables and Additional Data for observed Interactions 22

Coulomb–London–Pauli (CLP) Intermolecular Energy Calculations 25

References 33

Comparison between PGGE and EGE

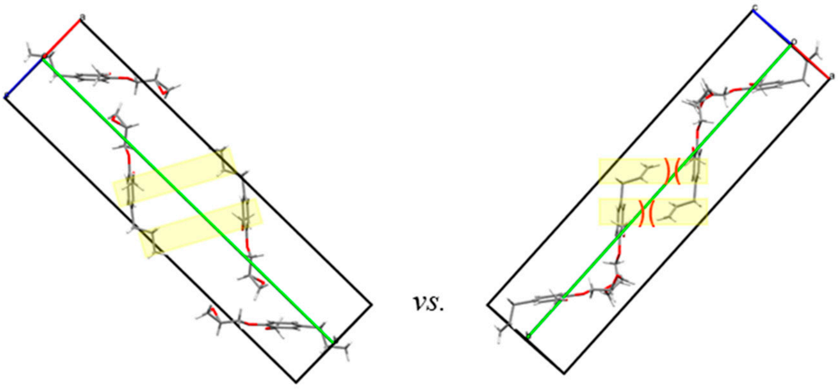

Figure S1. Comparison of PGGE and EGE respectively; the propyl side–arms for EGE are pointing towards the benzene ring of the adjacent molecule causing greater, possible steric repulsive interactions (highlighted in yellow with steric repulsion highlighted in red).

Materials and Methods

All the chemicals were commercially obtained from Merck® (Sigma–Aldrich®) or VWR® and used as received unless stated otherwise. Propyl guaiacol glycidyl ether, propyl guaiacol glycidyl ether dimer, eugenyl glycidyl ether and dihydroconiferyl alcohol glycidyl ether were prepared, *via* glycidylation, following previously reported protocols.[1,2] The Differential Scanning Calorimetry (DSC) analyses were recorded on a TA Instruments Discovery DSC instrument with the samples placed in T_{zero} pans with T_{zero} hermetic lids. The sample was cooled to -60 °C at 5 °C/min, equilibrated and held at this temperature for 2 minutes, heated to 70–200 °C at 5 °C/min (1st heating cycle), held at this temperature for 1 minutes, cooled to -60 °C at 5 °C/min, equilibrated and held at this temperature for 2 minutes and heated to 70–200 °C at 5 °C/min (2nd heating cycle). Unless stated otherwise, T_g, T_c and T_m values were determined from the 2nd heating cycle between -60 °C to 70–200 °C. As mentioned in the main text, the DCA sample was analyzed again at a slower cooling rate of 0.5 °C/min, however there was no change in the thermogram.

NMR spectroscopy was recorded on a Bruker AVIII 400 MHz or benchtop Magritek Spinsolve 80 MHz Ultra series Phosphor instrument and referenced to residual solvent signals such as $CHCl_3$. Phosphitylation and quantitative $^{31}P\ \{^1H\}$ NMR spectroscopy, using chromium acetylacetone, endo–*N*–hydroxy–5–norbornene–2,3–dicarboximide, pyridine, deuterated chloroform ($CDCl_3$) and 2–chloro–4,4,5,5–tetramethyl–1,3,2–dioxaphospholane, was conducted and analyzed following reported literature.[3–6] The Fourier–transform infrared spectroscopy (FT–IR) was performed using a Bruker Tensor 27 spectrometer and was measured using attenuated total reflectance mode (ATR–FTIR) under standard atmospheric conditions on a diamond crystal with a spectral range of 400–4000 cm^{-1}. The scan resolution was 4 cm^{-1} with 32 scans per sample and the spectra was baseline corrected. LC–MS was performed by Dr. J. Jordens, as part of the GOAL team at VITO N.V., on a Thermo Orbitrap Q Exactive coupled with a dionex UPLC–MS. A reversed phase LC measurement was performed using an Acquity BEH C18 100 x 2.1 mm 1.7 µm column with 10 mMH_4Ac as buffer A and CH_3CN as buffer B. With gradient: t = 0 min: 2% B, t = 2 min: 2% B, t = 10 min 100% B, t = 12 min 100% B, t = 12.1 min: 2% B, t = 15 min: 2% B. The column temperature was 60 °C and 5 µl was injected. Measurements were performed using ESI in positive and negative ion mode.

For propyl guaiacol glycidyl ether (PGGE), eugenyl glycidyl ether (EGE) and the methylene propyl guaiacol dimer (MPGD), suitable, single crystals were mounted on a LithoLoop and X–ray intensity data were collected on a SuperNova, Dual, Cu at home/near, Atlas diffractometer using Cu–K_α (λ = 1.54184 Å) radiation and recorded at 100(2) K (see *Table S1*). The images were interpreted and integrated with the program CrysAlisPro[7]. Using Olex2[8], the structures were solved by Intrinsic Phasing using the ShelXT structure solution program and refined by full-matrix least-squares on F^2 using the ShelXL program package[9,10]. Non-hydrogen atoms were anisotropically refined and the hydrogen atoms in the riding mode

with isotropic temperature factors fixed at 1.2 times U(eq) of the parent atoms (1.5 times for methyl groups). The structures were determined by Prof. K. Van Hecke.

For dihydroconiferyl alcohol (DCA), the propyl guaiacol dimer (PGD), the P–1 polymorph of EGE and the eugenol dimer (ED), suitable, single crystals were mounted on a Mitegen 200µm kapton loop and X–ray diffraction data were collected at room temperature (293(2)K) or 100(2)K with Cu–K_{α} (λ = 1.54184 Å) radiation (See *Table S1*) *via* ω–scans on a Rigaku R–Axis Rapid–S diffractometer, using CrystalClear 2.1.[11] Data reduction was done with HKL3000[12], the structure was solved with SHELXT–2018/3 and refined with SHELXL–2018/3[9,10], using the shelXle graphical interface.[13]

Hydrogen atoms for OH groups were geometrically calculated in idealized positions with O–H distances and H–bonding interactions freely refined (HFIX 148) and unnormalized. All other hydrogen atoms were refined using a riding model.

CCDC numbers 2359588–2359590 and 2361560–2361563 contain the supplementary crystallographic experimental data for this paper. These data can be obtained free of charge from The Cambridge Crystallographic Data Centre *via* www.ccdc.cam.ac.uk/structures.

CLP intermolecular energy calculations using the SCXRD solid–state structures were made with CrystalEplorer 3.0[14], based on B3LYP/6-31G(d,p) electron densities, but calibrated against a set of high–level ab initio calculations according to the methodology described in literature.[15] The molecule of interest was surrounded by a 3.8 Å radius sphere, and all molecules (partially) within this radius were completed. B3LYP/6-31G(d,p) electron densities were calculated with Tonto according to the crystal geometry from the input CIF file resulting from the refinement against the SCXRD data, and the resulting theoretical electron density grid was used directly for the CLP calculations (implemented with CrystalEplorer 3.0).[16] For crystal structures with two independent molecules in the unit cell, the process was repeated for the other independent molecule. Sublimation enthalpies were summed up from the individual

intermolecular contributions, and the result was divided by 2 to arrive at a figure per mole. If disorder was present in the structure, with the exception of the *P*–1 conformer of EGE, only the major configuration for every disorder present was taken into account. For EGE, we also removed the major conformer and kept the minor, but this led, as expected, to unrealistically small stabilization energies due to strong interference of the minor conformations with their neighbors. Images of interaction networks were made with CrystalExplorer 3.0.

Lignin Model Compound Synthesis and Characterization

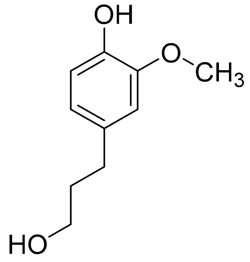

Dihydroconiferyl alcohol (DCA). This compound has previously been well reported in literature[4,17–20] and was prepared according to the following procedures.[18,20] ^{1}H NMR (400 MHz, $CDCl_3$) δ: 6.90–6.64 (m, 3H, Ar*H*), 5.71 (s, 1H, O*H*), 3.85 (s, 3H, OC*H*3), 3.67 (t, *J* = 6.4 Hz, 2H, CH2CH2C*H*2OH), 2.68–2.57 (m, 2H, C*H*2CH2CH2OH), 1.90–1.81 (m, 2H, CH2C*H*2CH2OH), 1.74 (s, 1H, O*H*). ^{13}C {^{1}H} NMR (101 MHz, $CDCl_3$) δ: 146.6 (Ar*C*–OCH3), 143.8 (Ar*C*–OH), 133.8 (Ar*C*–CH2CH2CH2OH), 121.0 (Ar*C*), 114.4 (Ar*C*), 111.2 (Ar*C*), 62.3 (CH2CH2*C*H2OH), 56.0 (O*C*H3), 34.5 (CH2*C*H2CH2OH), 31.8 (*C*H2CH2CH2OH). FT–IR is shown in *Figure S2*. DSC curve is shown in *Figure S9*. ^{31}P {^{1}H} NMR spectroscopy: found = 11.03 mmol total OH/g (5.42 mmol aliphatic OH/g and 5.61 mmol phenolic OH/g), theoretical = 11.0 mmol total OH/g. Recrystallisation and solid–state structure confirmed by using dichloromethane / petroleum ether to obtain crystals for single–crystal X–ray diffraction.

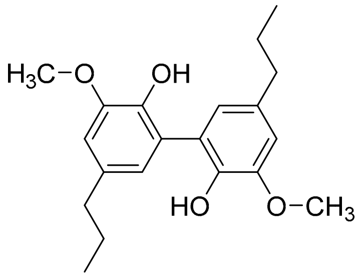

Propyl guaiacol dimer (PGD). This compound has been previously reported in literature[21,22] and was prepared according to the following literature using the enzyme Laccase (*Trametes versicolor*).[23–26] ^{1}H NMR (400 MHz, $CDCl_3$) δ: 6.79–6.67 (m, 4H, Ar*H*), 6.06 (s, 2H, O*H*), 3.92 (s, 6H, OC*H*3), 2.57 (t, *J* = 7.7 Hz, 4H, C*H*2CH2CH3), 1.66 (sextet, *J* = 7.4 Hz, 4H, CH2C*H*2CH3), 0.97 (t, *J* = 7.4 Hz, 6H, CH2CH2C*H*3). ^{13}C {^{1}H} NMR (101 MHz, $CDCl_3$) δ: 147.3 (Ar*C*–OCH3), 140.6 (Ar*C*–OH), 134.8 (Ar*C*–CH2CH2CH3), 124.6 (Ar*C*), 123.1 (Ar*C*), 110.8 (Ar*C*), 56.2 (O*C*H3), 38.0 (*C*H2CH2CH3), 24.9 (CH2*C*H2CH3), 14.0 (CH2CH2*C*H3). FT–IR is shown in *Figure S3*. DSC curve is shown in *Figure S10*. Recrystallisation and solid–state structure confirmed by using hot ethanol to obtain crystals for single–crystal X–ray diffraction.

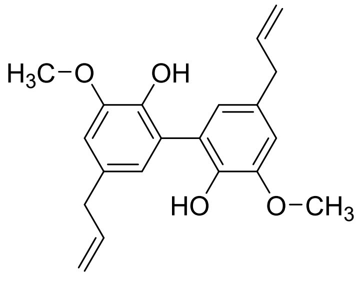

Eugenol dimer (ED) or dieugenol. This compound has been previously reported in literature and is commercially available.[22,27–29]. The compound was prepared according to the following literature using the enzyme Laccase (*Trametes versicolor*).[23–26] ^{1}H NMR (400 MHz, $CDCl_3$) δ: 6.78–6.67 (m, 4H, ArH), 6.05–5.91 (m, 4H, $CH_2C\mathit{H}=CH_2$ + OH), 5.15–5.02 (m, 4H, $CH_2CH=C\mathit{H}_2$), 3.92 (s, 6H, $OC\mathit{H}_3$), 3.37 (d, J = 6.7 Hz, 4H, $C\mathit{H}_2CH=CH_2$). ^{13}C {^{1}H} NMR (101 MHz, $CDCl_3$) δ: ArC–OCH_3 (147.4), 141.1 (ArC–OH), 137.8 ($CH_2\mathit{C}H=CH_2$), 132.1 (ArC–$CH_2CH=CH_2$), 124.5 (ArC), 123.3 (ArC), 115.9 ($CH_2CH=\mathit{C}H_2$), 110.8 (ArC), 56.2 ($O\mathit{C}H_3$), 40.1 ($\mathit{C}H_2CH=CH_2$). FT–IR is shown in *Figure S4*. DSC curve is shown in *Figure S11*. Recrystallisation and solid–state structure confirmed by using hot ethanol to obtain crystals for single–crystal X–ray diffraction.

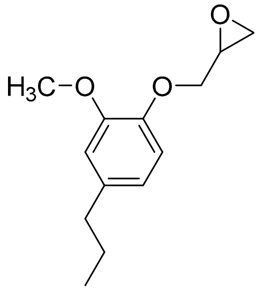

Propyl guaiacol glycidyl ether (PGGE). The compound was prepared following a previously reported epoxidation protocol[2] and similar epoxidized guaiacol classed compounds are reported in literature.[30–33] ^{1}H NMR (400 MHz, $CDCl_3$) δ: 6.88–6.64 (m, 3H, ArH), 4.19 (dd, J = 11.3, 3.6 Hz, 1H, $C\mathit{H}_2$–glycidyl ether), 4.01 (dd, J = 11.4, 5.5 Hz, 1H, $C\mathit{H}_2$–glycidyl ether), 3.85 (s, 3H, $OC\mathit{H}_3$), 3.39–3.34 (m, 1H, CH of the glycidyl ether), 2.87 (dd, J = 5.0, 4.0 Hz, 1H, $C\mathit{H}_2$ of the glycidyl ether), 2.72 (dd, J = 5.0, 2.6 Hz, 1H, $C\mathit{H}_2$ of the glycidyl ether), 2.56–2.48 (m, 2H, $C\mathit{H}_2CH_2CH_3$), 1.61 (sextet, J = 14.7, 7.3 Hz, 2H, $CH_2C\mathit{H}_2CH_3$), 0.93 (t, J = 7.3 Hz, 3H, $CH_2CH_2C\mathit{H}_3$). ^{13}C {^{1}H} NMR (101 MHz, $CDCl_3$) δ: 149.5 (ArC–OCH_3), 146.1 (ArC–OCH_2–glycidyl ether), 136.8 (ArC–$CH_2CH_2CH_3$), 120.4 (ArC), 114.5 (ArC), 112.5 (ArC), 70.6 (ArC–$O\mathit{C}H_2$–glycidyl ether), 56.0 ($O\mathit{C}H_3$), 50.4 (CH of the glycidyl ether), 45.1 ($\mathit{C}H_2$ of the glycidyl ether), 37.8 ($\mathit{C}H_2CH_2CH_3$), 24.8 ($CH_2\mathit{C}H_2CH_3$), 13.9 ($CH_2CH_2\mathit{C}H_3$). LC–MS: Calcd product = [$C_{13}H_{18}O_3$], found = [$C_{13}H_{18}O_3$]. FT–IR is shown in *Figure S5*. DSC curve is shown in *Figure S12*. Elemental analysis: Calcd for $C_{13}H_{18}O_3$ (found): C, 70.24 (68.28); H, 8.16 (8.05); N, 0.00

(<0.3). Recrystallisation and solid–state structure confirmed by using hot toluene to obtain crystals, that were stable at room temperature, for single–crystal X–ray diffraction.

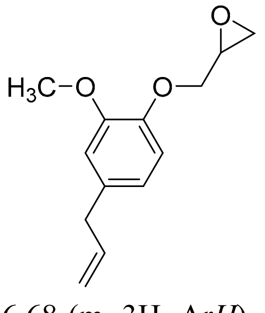

Eugenyl glycidyl ether (EGE). These compound, and similar compounds, have been previously reported in literature.[30–32] In this instance, it was prepared according to the following a previously reported epoxidation protocol.[2] ^{1}H NMR (400 MHz, $CDCl_3$) δ: 6.89–6.68 (m, 3H, Ar*H*), 5.95 (ddt, J = 16.8, 10.1, 6.7 Hz, 1H, CH_2C*H*=CH_2), 5.13–4.99 (m, 2H, CH_2CH=C*H*$_2$), 4.20 (dd, J = 11.4, 3.6 Hz, 1H, C*H*$_2$–glycidyl ether), 4.02 (dd, J = 11.4, 5.5 Hz, 1H, C*H*$_2$–glycidyl ether), 3.85 (s, 3H, OC*H*$_3$), 3.37 (m, 1H, C*H* of the glycidyl ether), 3.33 (d, J = 6.7 Hz, 2H, C*H*$_2$CH=CH_2), 2.88 (dd, J = 5.0, 4.0 Hz, 1H, C*H*$_2$ of the glycidyl ether), 2.72 (dd, J = 5.0, 2.6 Hz, 1H, C*H*$_2$ of the glycidyl ether). ^{13}C {^{1}H} NMR (101 MHz, $CDCl_3$) δ: 149.7 (Ar*C*–OCH_3), 146.4 (Ar*C*–OCH_2–glycidyl ether), 137.6 (CH_2*C*H=CH_2), 134.0 (Ar*C*–CH_2CH=CH_2), 120.6 (Ar*C*), 115.8 (CH_2CH=*C*H$_2$), 114.6 (Ar*C*), 112.6 (Ar*C*), 70.6 (ArC–O*C*H$_2$–glycidyl ether), 56.0 (O*C*H$_3$), 50.4 (*C*H of the glycidyl ether), 45.1 (*C*H$_2$ of the glycidyl ether), 39.9 (*C*H$_2$CH=CH_2). LC–MS: Calcd product = [$C_{13}H_{16}O_3$], found = [$C_{13}H_{16}O_3$]. FT–IR is shown in *Figure S6*. DSC curve is shown in *Figure S13*. Elemental analysis: Calcd for $C_{13}H_{16}O_3$ (found): C, 70.89 (65.12); H, 7.32 (7.80); N, 0.00 (<0.3). Recrystallisation achieved by using toluene to obtain crystals with the $P2_1/n$ space group and using n-propanol to obtain crystals with the *P*-1 space group. These crystals were less stable at room temperature and had to be stored in a fridge to prevent melting. The solid–state structure was confirmed *via* placing the crystal on ice before mounting for single–crystal X–ray diffraction.

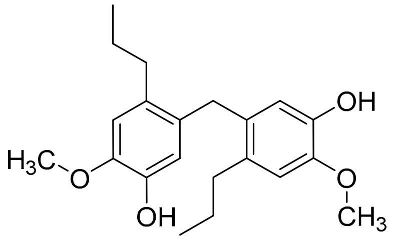

Methylene propyl guaiacol dimer (MPGD). This compound has previously been reported in literature and was prepared according to the following procedure.[34] ^{1}H NMR (80 MHz, $CDCl_3$) δ: 6.67 (s, 2H, Ar*H*), 6.48 (s, 2H, Ar*H*), 5.34 (s, 2H,

OH), 3.87 (s, 6H, OCH_3), 3.78 (s, 2H, Ar–CH_2–Ar), 2.49 (t, J = 7.2 Hz, 4H, CH_2CH$_2$CH$_3$), 1.88–1.24 (m, 4H), 0.94 (t, J = 7.1 Hz, 6H, CH$_2$CH$_2$CH_3). Consistent with that reported in literature.[34] Recrystallisation and solid–state structure confirmed by using diethyl ether / heptane to obtain crystals for single–crystal X–ray diffraction. DSC curve is shown in *Figure S14*. Melting point (measured *via* the DSC) = 81 °C and consistent with literature.[34]

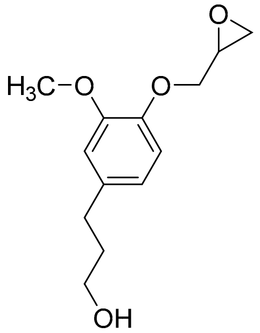

Dihydroconiferyl alcohol glycidyl ether (DCAGE). The compound was prepared from the synthesized dihydroconiferyl alcohol (DCA) following a previously reported epoxidation protocol[2] and similar epoxidized guaiacol classed compounds are reported in literature.[30–33] ^{1}H NMR (400 MHz, CDCl$_3$) δ: 6.92–6.65 (m, 3H, ArH), 4.21 (dd, J = 11.4, 3.6 Hz, 1H, CH_2–glycidyl ether), 4.02 (dd, J = 11.4, 5.5 Hz, 1H, CH_2–glycidyl ether), 3.86 (s, 3H, OCH_3), 3.73–3.63 (m, 2H, CH$_2$CH$_2$CH_2OH), 3.42–3.33 (m, 1H, CH of the glycidyl ether), 2.89 (dd, J = 4.9, 4.1 Hz, 1H, CH_2 of the glycidyl ether), 2.73 (dd, J = 4.9, 2.6 Hz, 1H, CH_2 of the glycidyl ether), 2.69–2.57 (m, 2H, CH_2CH$_2$CH$_2$OH), 1.94–1.82 (m, 2H, CH$_2$CH_2CH$_2$OH). ^{13}C {^{1}H} NMR (101 MHz, CDCl$_3$) δ: 149.5 (ArC–OCH$_3$), 146.2 (ArC–OCH$_2$–glycidyl ether), 135.9 (ArC–CH$_2$CH$_2$CH$_2$OH), 120.4 (ArC), 114.6 (ArC), 112.5 (ArC), 70.6 (ArC–OCH$_2$–glycidyl ether), 62.1 (CH$_2$CH$_2$$CH_2$OH), 55.9 (O$CH_3$), 50.4 ($C$H of the glycidyl ether), 45.1 (CH$_2$ of the glycidyl ether), 34.3 (CH$_2$$CH_2CH_2$OH), 31.8 ($CH_2CH_2CH_2$OH). In both the ^{1}H NMR and ^{13}C {^{1}H} NMR spectra was also observed a small amount of minor aliphatic resonance signals related to residual ethyl acetate solvent and also most likely DCA synthesis and/or undesired glycidylation side–products. Phosphitylation and quantitative ^{31}P {^{1}H} NMR (32 MHz, CDCl$_3$): found = 4.87 mmol aliphatic OH/g, theoretical = 4.23 mmol aliphatic OH/g. LC–MS: Calcd product = [$C_{13}H_{18}O_4$], found = [$C_{13}H_{18}O_4$]. FT–IR is shown in *Figure S7*. DSC curve is shown in *Figure S15*. Elemental analysis: Calcd for

$C_{13}H_{18}O_4$ (found): C, 65.53 (68.09); H, 7.61 (7.46); N, 0.00 (<0.3). Attempts at recrystallisation to obtain crystals for single–crystal X–ray diffraction were unsuccessful.

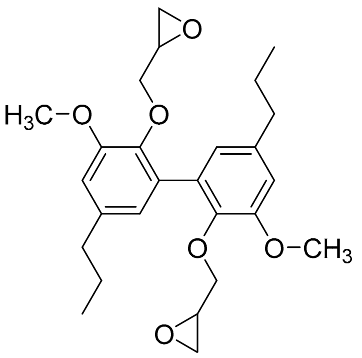

Propyl guaiacol dimer diglycidyl ether (PGD diepoxide). The compound was prepared from the synthesized propyl guaiacol dimer (PGD) following a previously reported epoxidation protocol for DSC analysis.[2] Similar diglycidyl ether dimer compounds are reported in literature.[28,35–38] ^{1}H NMR (80 MHz, $CDCl_3$) δ: 6.67 (m, 4H, Ar*H*), 4.29–3.64 (m, 10H, OC*H*$_3$ + C*H*$_2$–glycidyl ether), 3.18–2.90 (m, 2H, C*H* of the glycidyl ether), 2.74–2.30 (m, 8H, C*H*$_2$CH$_2$CH$_3$ + C*H*$_2$ of the glycidyl ether), 1.90–1.40 (m, 4H, CH$_2$C*H*$_2$CH$_3$), 0.95 (t, J = 7.3 Hz, 6H, CH$_2$CH$_2$C*H*$_3$). DSC curve is shown in *Figure S16*. Elemental analysis: Calcd for $C_{26}H_{34}O_6$ (found): C, 70.56 (69.3); H, 7.74 (8.0); N, 0.00 (<0.1).

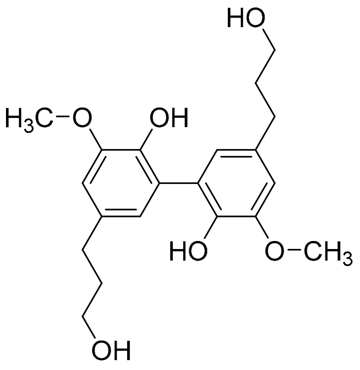

Dihydroconiferyl alcohol dimer (DCA dimer). The compound was prepared as part of a three–step synthesis: 1) Enzymatic acetate protection of the aliphatic OH group using enzyme Novozymes 51003 (*Aspergillus Oryzae*). 2) Dimerization using the enzyme Laccase (*Trametes versicolor*). 3) Deprotection of the acetate group. This was following reported literature.[23–26,39] ^{1}H NMR (400 MHz, DMSO–d^6) δ: 8.08 (s, 2H, ArO*H*), 6.70 (d, J = 2.0 Hz, 2H, Ar*H*), 6.50 (d, J = 2.0 Hz, 2H, Ar*H*), 4.38 (t, J = 5.2 Hz, 2H, CH$_2$CH$_2$C*H*$_2$OH), 3.76 (s, 6H, OC*H*$_3$), 3.40–3.35 (m, 4H, C*H*$_2$CH$_2$CH$_2$OH), 1.71–1.59 (m, 4H, CH$_2$C*H*$_2$CH$_2$OH). ^{13}C {^{1}H} NMR (101 MHz, DMSO–d^6) δ: 147.6 (Ar*C*–OCH$_3$), 141.4 (Ar*C*–OH), 132.2 (Ar*C*–CH$_2$CH$_2$CH$_2$OH), 125.9 (Ar*C*), 122.7 (Ar*C*), 110.7 (Ar*C*), 60.2 (CH$_2$CH$_2$*C*H$_2$OH), 55.8 (O*C*H$_3$), 34.6 (CH$_2$*C*H$_2$CH$_2$OH), 31.3 (*C*H$_2$CH$_2$CH$_2$OH). FT–IR is shown in *Figure S8*. DSC curve is shown in *Figure S17*.

IR Spectra of Lignin Model Compounds

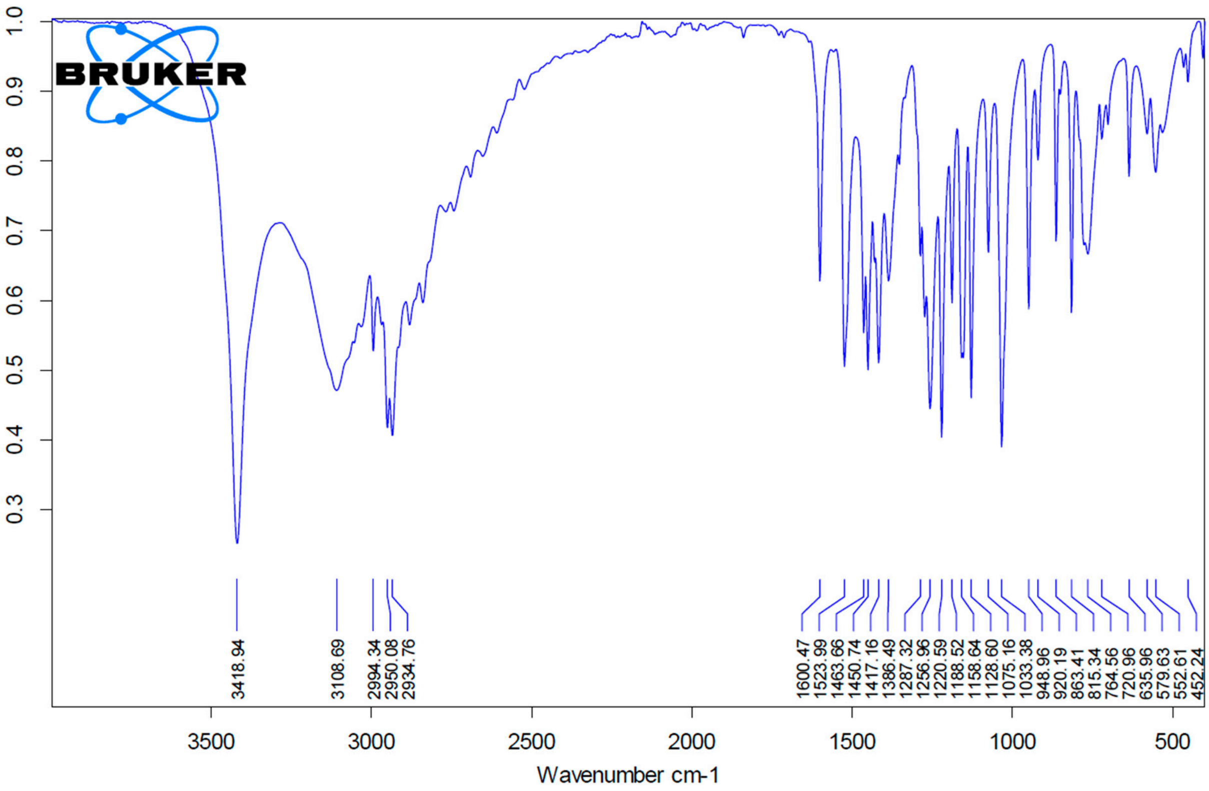

Figure S2. FT–IR of dihydroconiferyl alcohol (DCA).

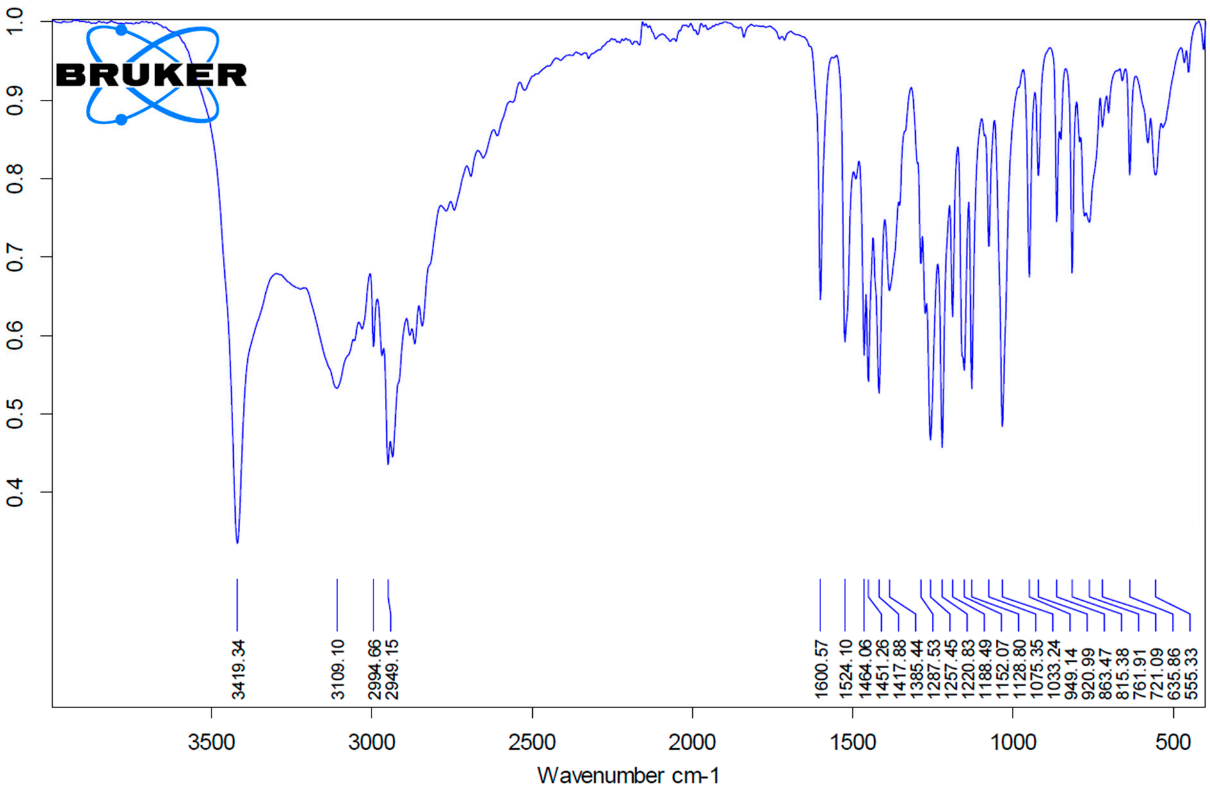

Figure S3. FT–IR of propyl guaiacol dimer (PGD).

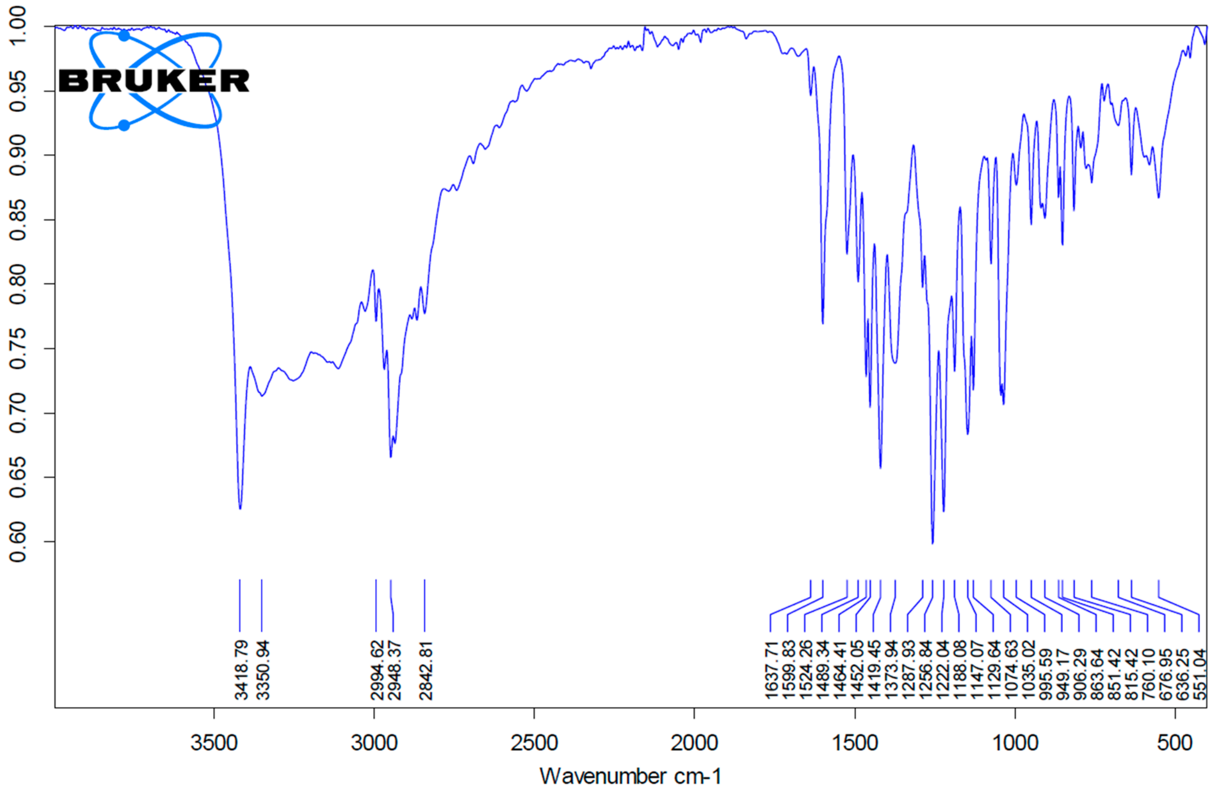

Figure S4. FT–IR of eugenol dimer (ED).

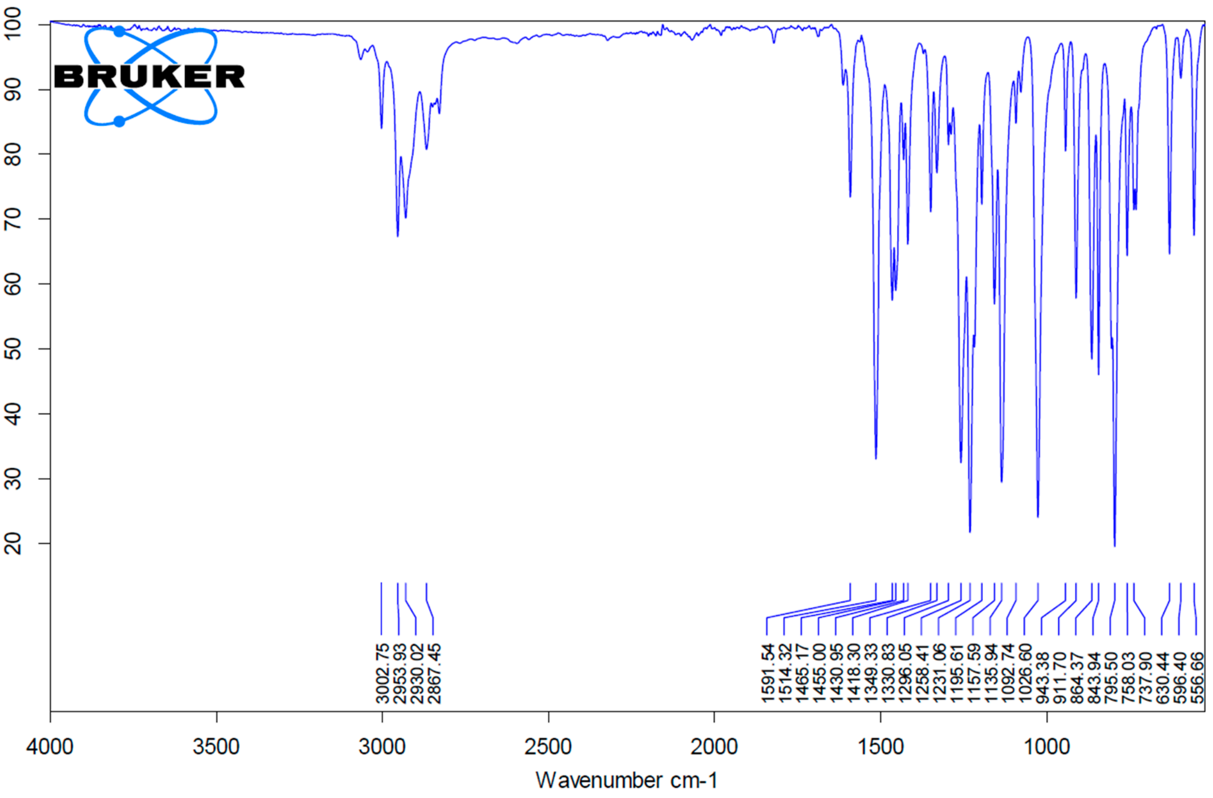

Figure S5. FT–IR of propyl guaiacol glycidyl ether (PGGE).

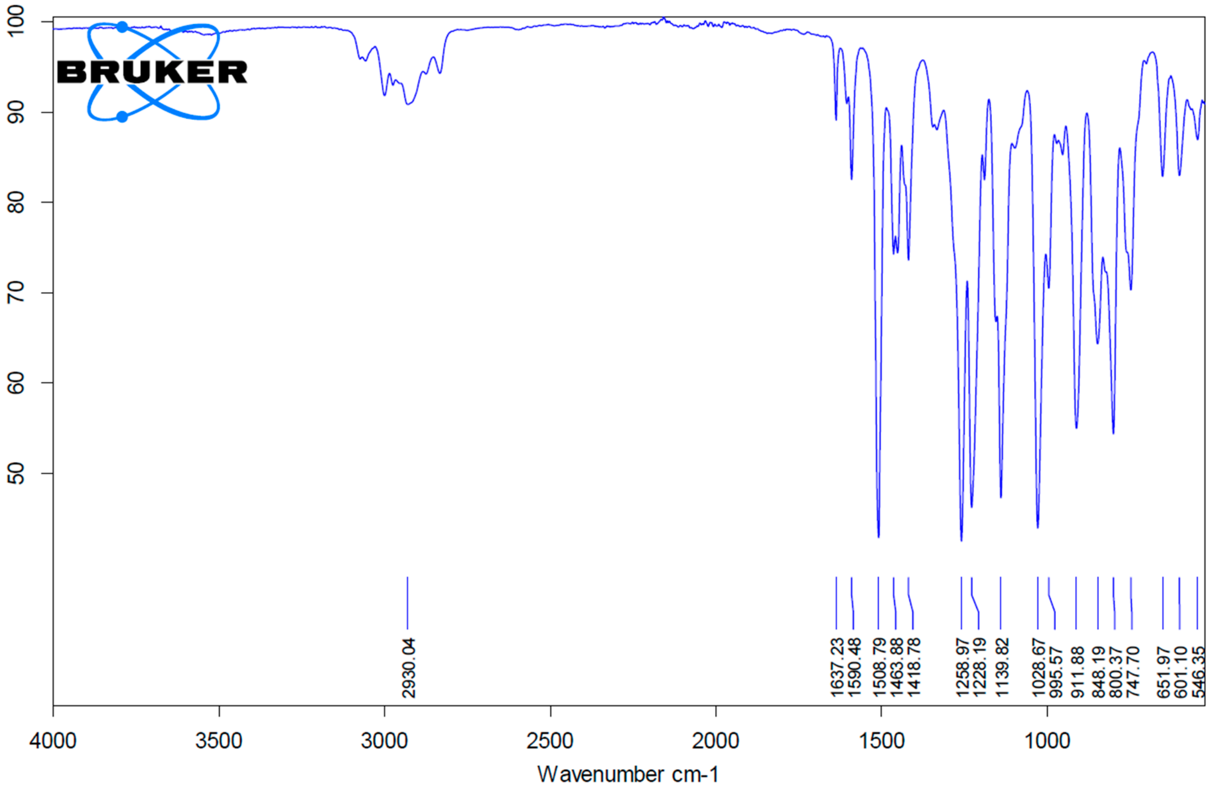

Figure S6. FT–IR of eugenyl glycidyl ether (EGE).

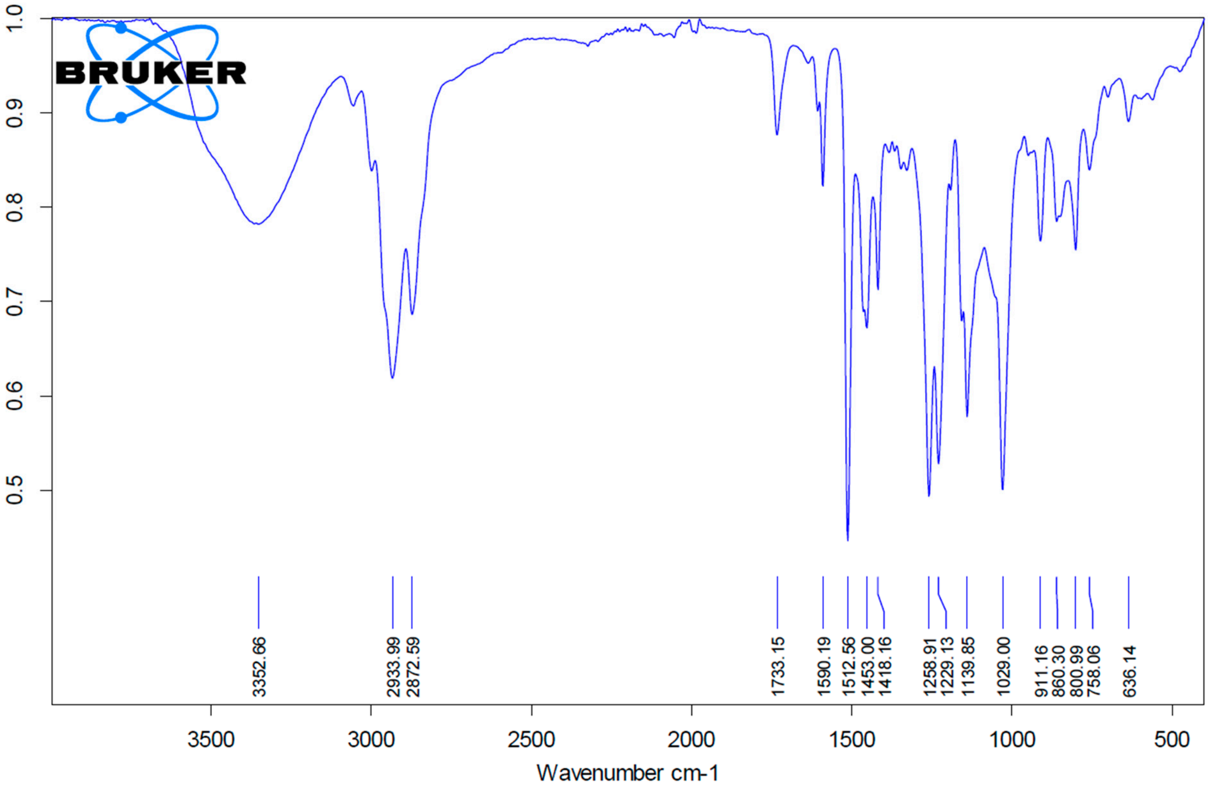

Figure S7. FT–IR of dihydroconiferyl alcohol glycidyl ether (DCAGE).

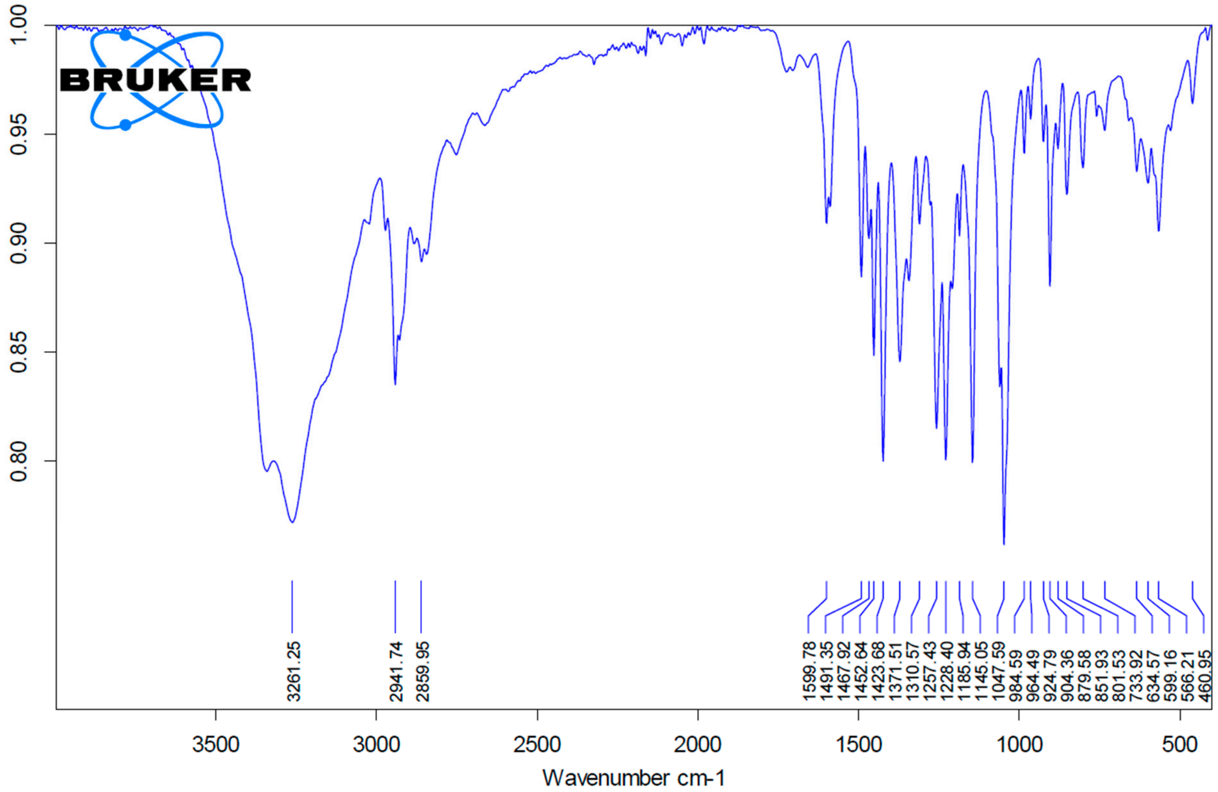

Figure S8. FT–IR of dihydroconiferyl alcohol dimer (DCA dimer).

DSC Curves of Lignin Model Compounds

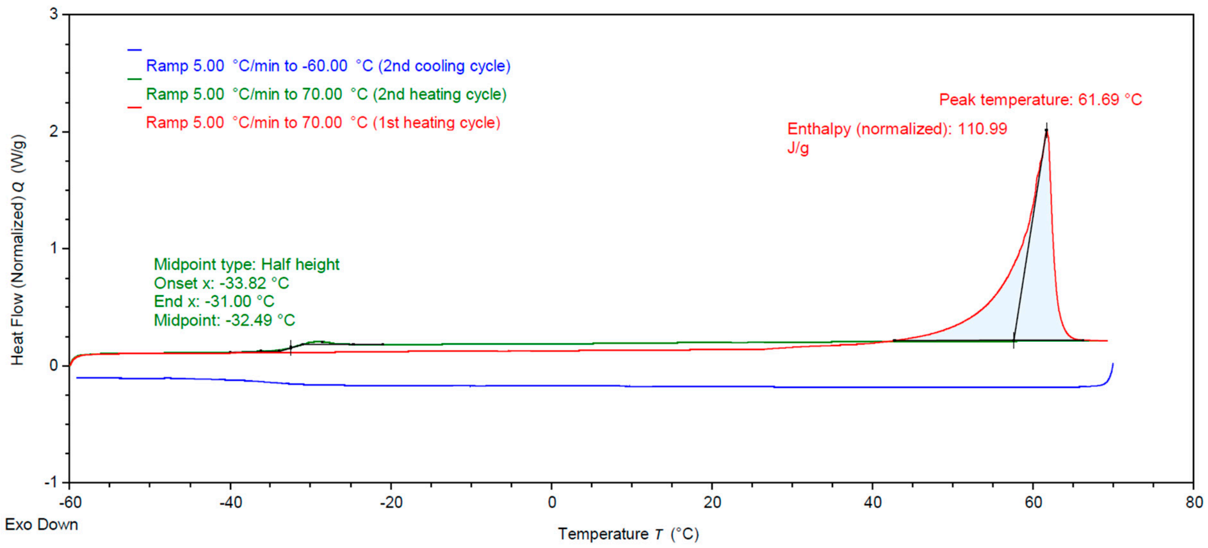

Figure S9. DSC curve of dihydroconiferyl alcohol (DCA).

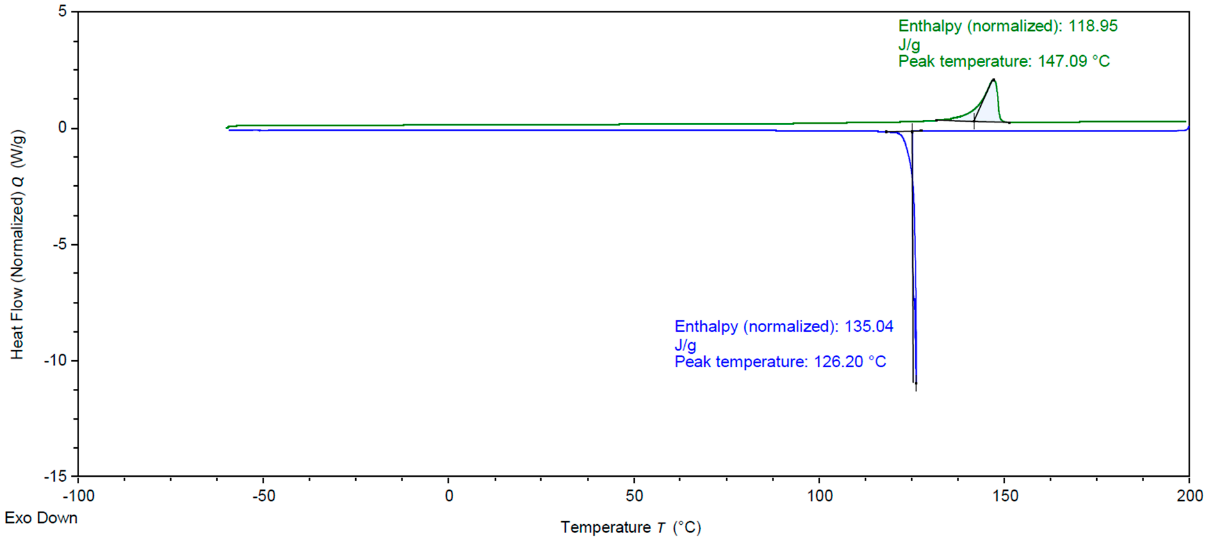

Figure S10. DSC curve of propyl guaiacol dimer (PGD).

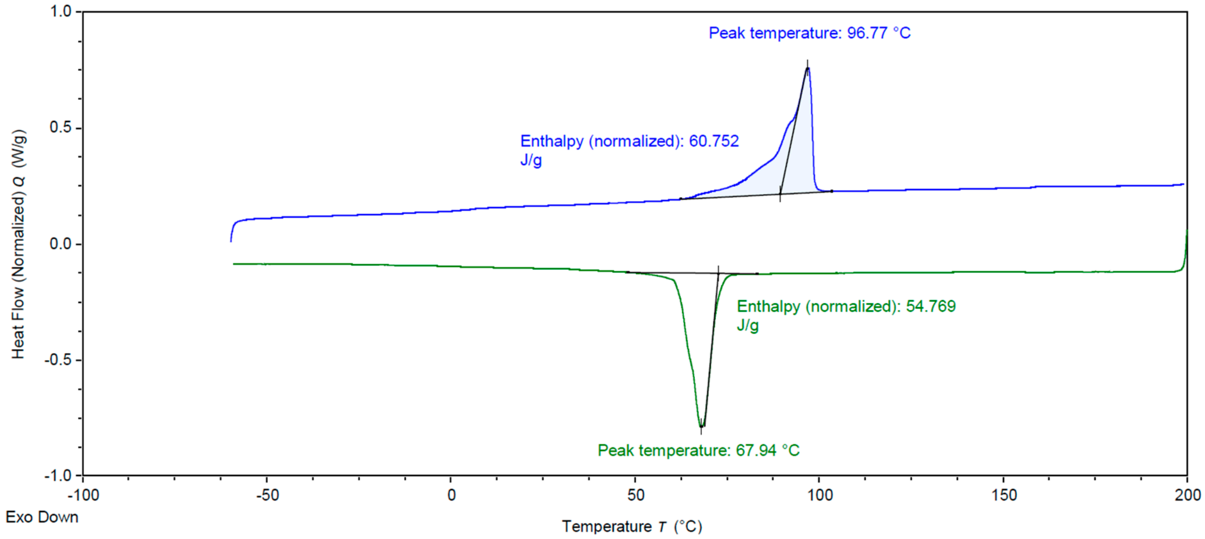

Figure S11. DSC curve of eugenol dimer (ED).

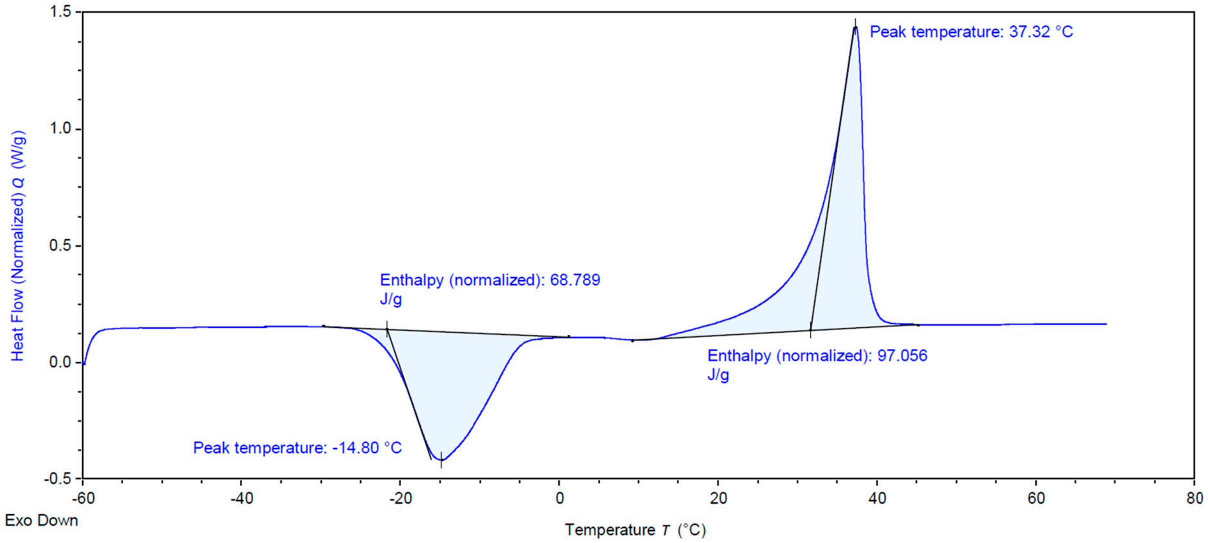

Figure S12. DSC curve of propyl guaiacol glycidyl ether (PGGE).

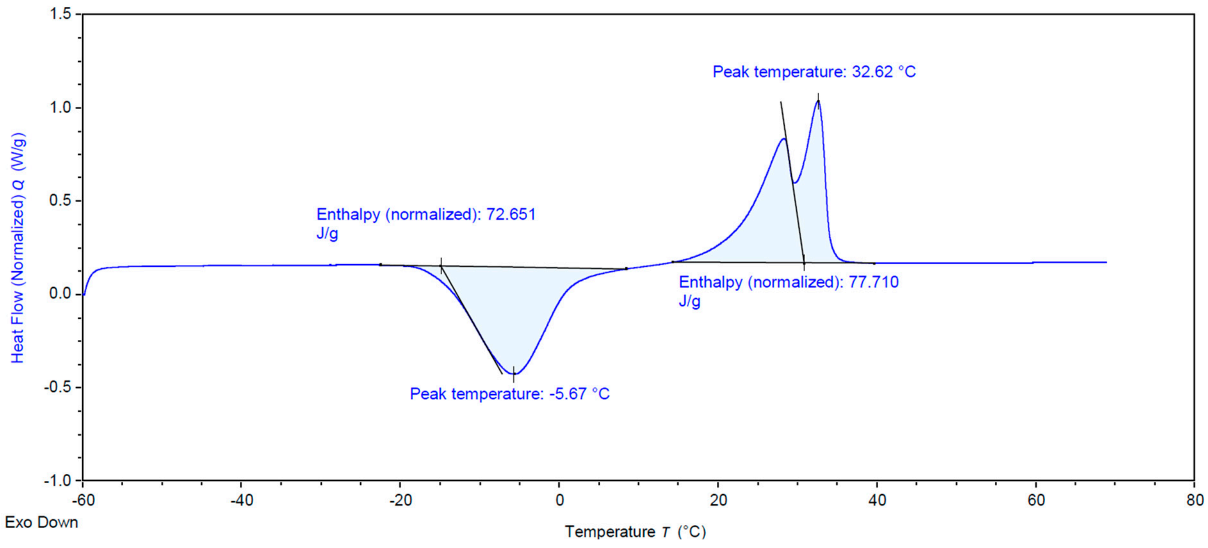

Figure S13. DSC curve of eugenyl glycidyl ether (EGE).

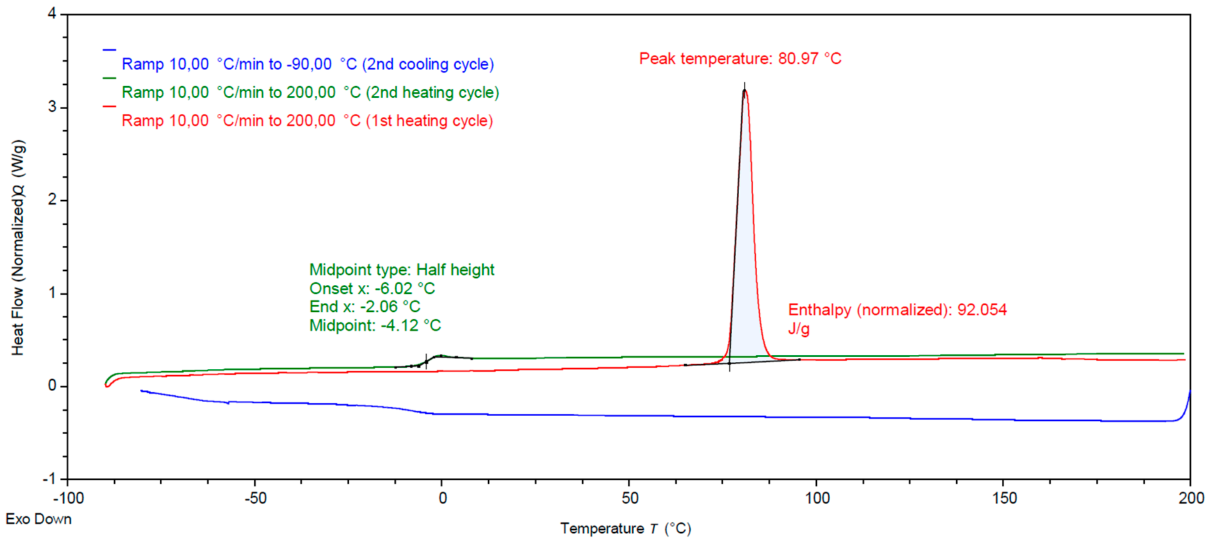

Figure S14. DSC curve of methylene propyl guaiacol dimer (MPGD).

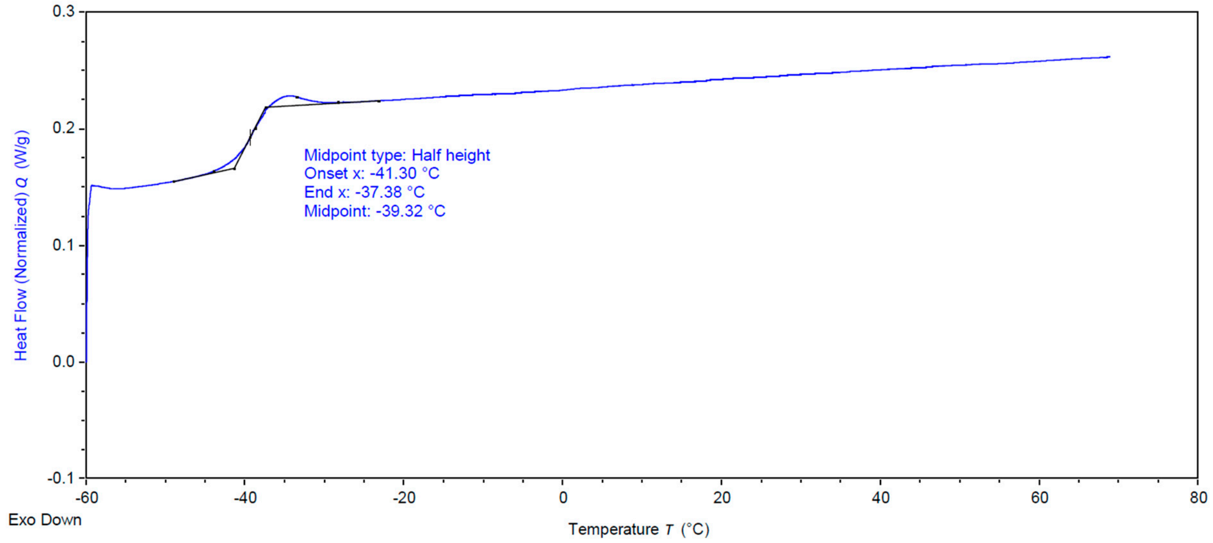

Figure S15. DSC curve of dihydroconiferyl alcohol glycidyl ether (DCAGE).

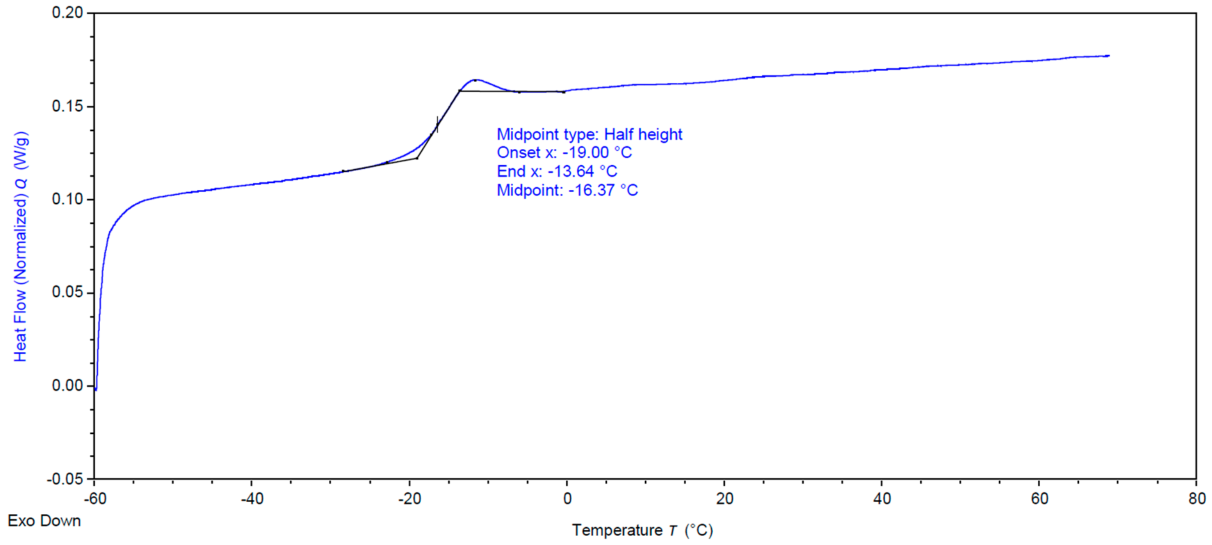

Figure S16. DSC curve of propyl guaiacol dimer diglycidyl ether (PGD diepoxide).

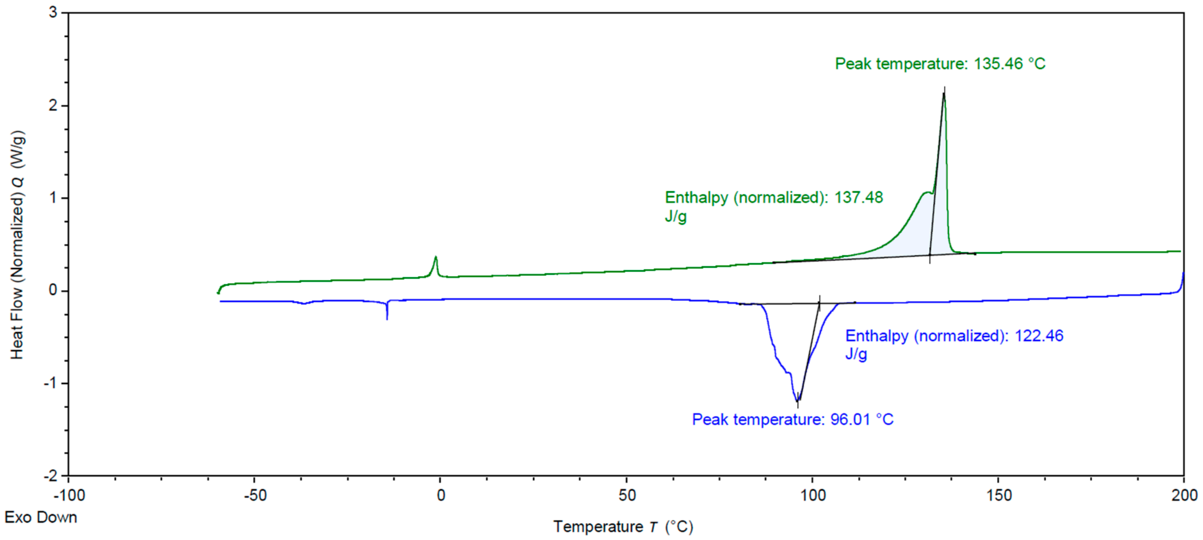

Figure S17. DSC curve of dihydroconiferyl alcohol dimer (DCA dimer).

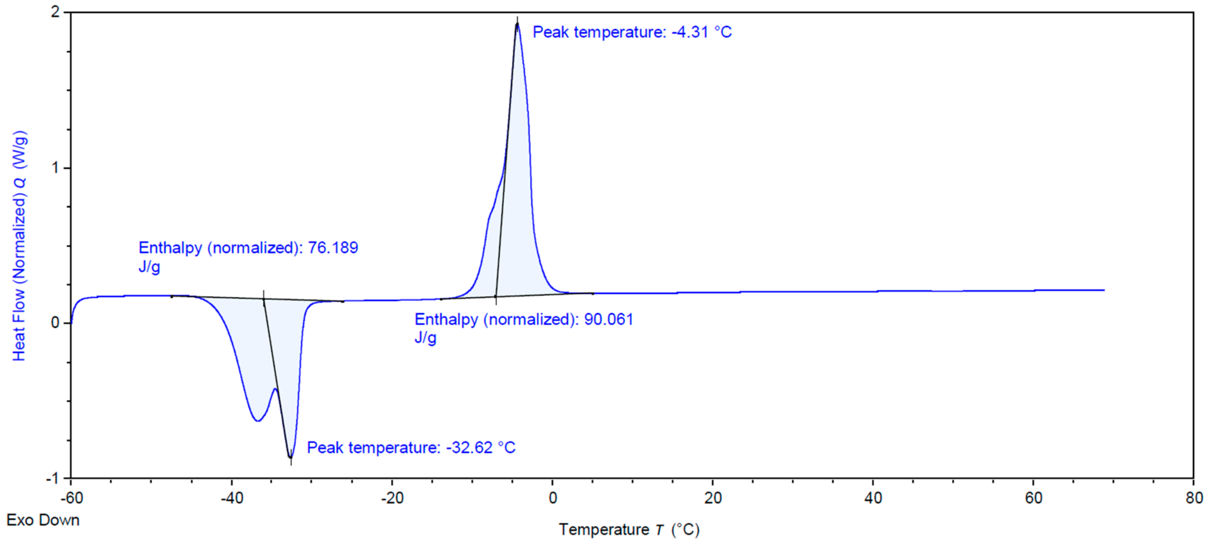

Figure S18. DSC curve of commercial propyl guaiacol (PG).

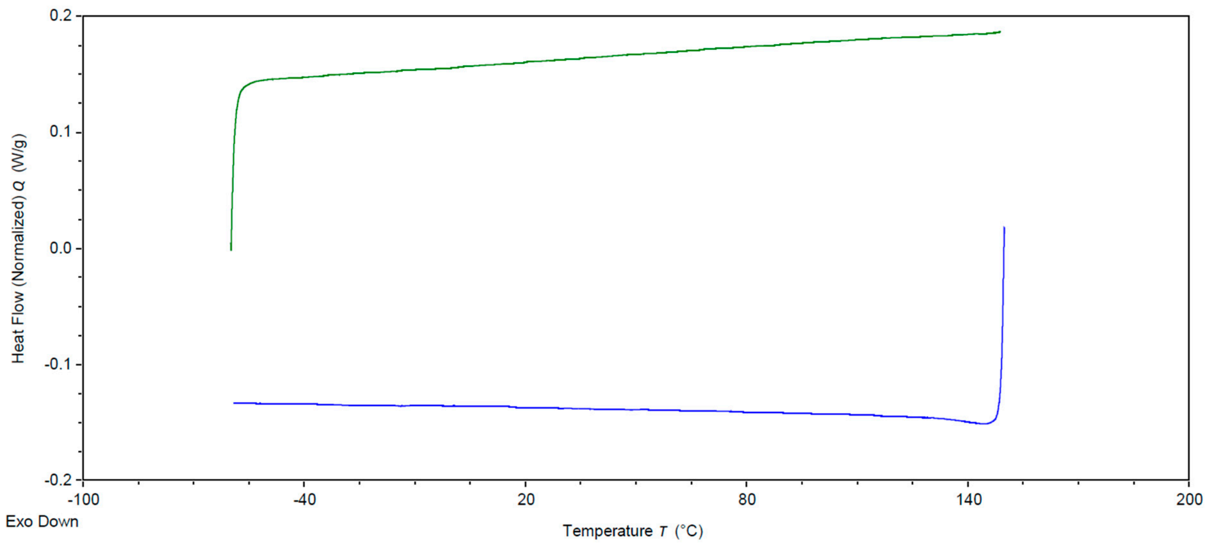

Figure S19. DSC curve of commercial eugenol (E)**.**

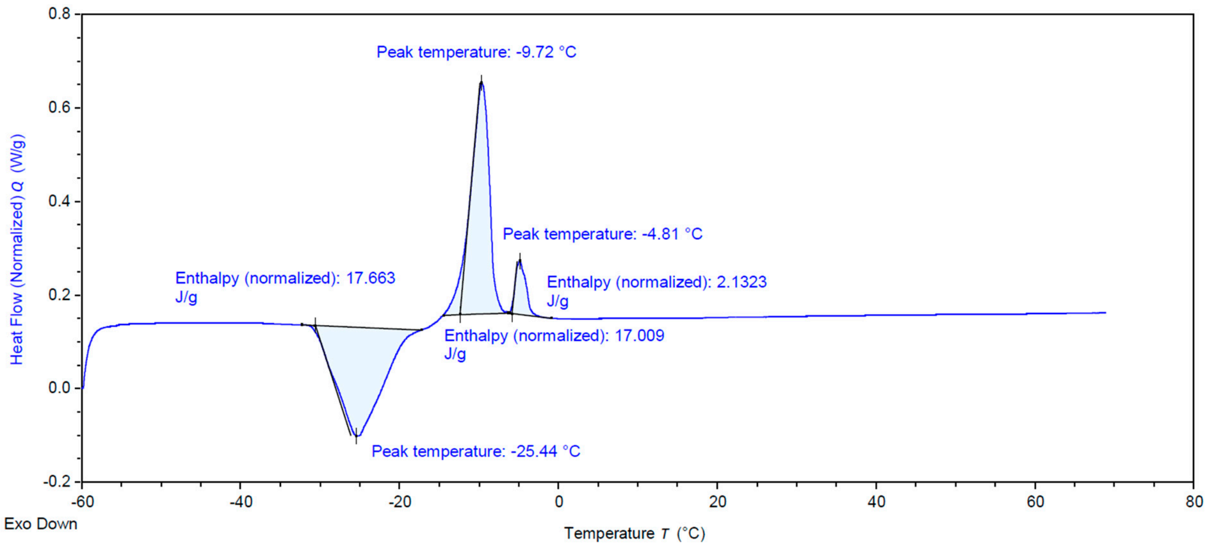

Figure S20. DSC curve of commercial phenyl glycidyl ether (PGE).

Crystallographic Tables and Additional Data for Observed Interactions

Table S1. Data collection and refinement statistics of all solid–state X–ray structures obtained in this study.

Compound reference	DCA	PGGE	EGE	EGE	PGD	ED	MPGD
Chemical formula	$C_{20}H_{28}O_6$	$C_{13}H_{18}O_3$	$C_{13}H_{16}O_3$	$C_{13}H_{16}O_3$	$C_{20}H_{26}O_4$	$C_{20}H_{22}O_4$	$C_{21}H_{28}O_4$
Formula Mass	364.44	222.27	220.26	220.26	330.42	326.39	344.43
Crystal system	Orthorhombic	Monoclinic	Monoclinic	Triclinic	Monoclinic	Triclinic	Orthorhombic
Space group	*Pbca*	$P2_1/n$	$P2_1/n$	*P*-1	$P2_1/n$	*P*-1	*Pbcn*
a/Å	16.8772(8)	4.69745(4)	4.57278(5)	4.800(1)	12.1330(8)	8.9744(10)	14.29790(10)
b/Å	6.6647(3)	32.1516(2)	31.8895(3)	9.808(2)	9.0268(5)	12.4696(17)	8.83350(10)
c/Å	17.2045(7)	7.88843(8)	7.85762(9)	12.398(2)	33.6388(16)	16.4182(18)	30.3232(3)
α/°	90	90	90	87.37(2)	90	79.393(9)	90
β/°	90	98.0546(9)	96.3333(10)	82.33(2)	99.617(4)	81.097(10)	90
γ/°	90	90	90	82.20(2)	90	88.283(10)	90
Unit cell volume/Å^3	1935.47(14)	1179.641(17)	1138.83(2)	572.9(2)	3632.4(4)	1784.2(4)	3829.84(6)
Temperature/K	293(2)	100(2)	100(2)	100(2)	293(2)	293(2)	100(2)
No. of formula units per unit cell, *Z*	4	4	4	2	8	4	8
Radiation type	Cu Kα	Cu Kα	Cu Kα	Cu Kα	Cu Kα	Cu Kα	Cu Kα
No. of reflections measured	14555	33721	32507	6794	30635	31959	32143
No. of independent reflections	1880	2385	2304	1934	6903	6203	3864
R_{int}	0.0385	0.0354	0.0620	0.053	0.0488	0.0526	0.0486
Final R_1 values $[(I > 2\sigma(I)]$	0.0388	0.0377	0.0426	0.087	0.0555	0.0583	0.0379
Final wR_2 values $[(I > 2\sigma(I)]$	0.1042	0.0928	0.1165	0.2298	0.1553	0.1696	0.1018
Final R_1 values (all data)	0.0398	0.0429	0.0488	0.1013	0.0728	0.0769	0.0451
Final wR_2 values (all data)	0.1054	0.0969	0.1236	0.2516	0.1738	0.1963	0.1086

Eugenyl glycidyl ether (EGE)

C–H···*Cg*(π) intermolecular interactions (Å, °) (*Figure 4b* of the main text)

X–H···*Cg*	H···*Cg*	Symmetry code
C7–H7A···*Cg*2	2.90	1+x, y, z

*Cg*2 the centroid of the C1–C6 ring.

Hydrogen–bond geometry (Å, °) (*Figure 4b* of the main text)

D–H···*A*	H···*A*	*D*–H···*A*
C9-H9A···O2	2.623 Å	158.26°

Symmetry code: 1/2+x, 3/2-y, 1/2+z

Eugenol dimer (ED)

Hydrogen–bond geometry (Å, °)

D–H···*A*	Type	*D*–H	H···*A*	*D*···*A*	*D*–H···*A*
O1–H1···O2	Intramolecular	0.85(3)	2.17(3)	2.638(3)	114(2)
O1–H1···O21^{i}	Intermolecular	0.85(3)	2.18(3)	2.964(2)	152(2)
O1–H1···O22^{i}	Intermolecular	0.85(3)	2.45(3)	2.965(3)	120(3)
O31–H1A···O11^{ii}	Intermolecular	0.84(3)	2.24(3)	2.974(2)	147(2)
O31–H1A···O12^{ii}	Intermolecular	0.84(3)	2.39(3)	2.951(3)	125(3)
O31–H1A···O32	Intramolecular	0.84(3)	2.21(3)	2.655(3)	113(2)
O21–H1B···O31	Intramolecular	0.86(4)	1.90(4)	2.694(2)	153(2)
O11–H11···O1	Intramolecular	0.84(4)	1.88(4)	2.660(2)	155(2)

Symmetry codes: (i) 1+x,y,-1+z, (ii) x,y,1+z

C–H···*Cg*(π) intermolecular interactions (Å, °)

X–H···*Cg*	H···*Cg*	X···*Cg*	X–H···*Cg*
C40–H40A···*Cg*4^{i}	2.99	3.651(4)	127

Symmetry codes: (i): 2-x,-y,1-z. *Cg*4 the centroid of the C11–C16 ring.

$Cg(\pi)\cdots Cg(\pi)$ intermolecular interactions (Å, °)

Cg(I)···*Cg*(J)	*Cg*···*Cg*	α	β	γ	*Cg*(I) perpendicular	*Cg*(J) perpendicular
Cg(2)···*Cg*(4)^{i}	5.6488(16)	81.70(12)	21.5	85.1	0.4785(10)	5.2564(10)
Cg(3)···*Cg*(1)^{ii}	5.5484(17)	81.42(13)	17.7	89.2	0.0818(10)	5.2868(11)
Cg(4)···*Cg*(2)^{iii}	5.8196(16)	81.70(12)	33.7	89.1	0.0944(10)	4.8416(10)

Symmetry codes: (i): 1-x,-y,1-z, (ii) 1-x,1-y,1-z, (iii) 2-x,-y,1-z. *Cg*1 the centroid of the C21–C26 ring, *Cg*2 the centroid of the C31–C36 ring, *Cg*3 the centroid of the C1–C6 ring and *Cg*4 the centroid of the C11–C16 ring. The Fractional Atomic Coordinates for the *Cg* rings are below.

Fractional Atomic Coordinates of the *Cg* centroids.

Cg(X)	*x*	*y*	*z*
*Cg*1	0.35288(13)	0.43164(9)	0.79603(7)
*Cg*2	0.73410(11)	0.21543(8)	0.78196(6)
*Cg*3	1.11743(12)	0.27095(8)	0.22970(6)
*Cg*4	0.74147(11)	0.07065(8)	0.21930(7)

Coulomb–London–Pauli (CLP) Intermolecular Energy Calculations

DCA

N	Symop	R	Electron Density	E_ele	E_pol	E_dis	E_rep	E_tot	
2	x, y, z	6,66	B3LYP/6-31G(d,p)	-4,6	-0,7	-15,7	8	-14,1	-28,2
1	-x, -y, -z	5,48	B3LYP/6-31G(d,p)	-112,5	-25,9	-35,5	144,8	-79,6	-79,6
2	-x+1/2, y+1/2, z	5,51	B3LYP/6-31G(d,p)	-6,7	-1,6	-32,1	19,5	-24,1	-48,2
2	x, -y+1/2, z+1/2	8,61	B3LYP/6-31G(d,p)	-35,4	-3	-18,1	40,7	-30,3	-60,6
1	-x, -y, -z	5,21	B3LYP/6-31G(d,p)	-8,8	-1,6	-28	13,5	-26,6	-26,6
2	-x, y+1/2, -z+1/2	10,98	B3LYP/6-31G(d,p)	-2,2	-0,3	-2,3	0,2	-4,5	-9
2	-x+1/2, -y, z+1/2	10,15	B3LYP/6-31G(d,p)	-0,1	-0,2	-4,7	1,1	-3,7	-7,4
2	x, -y+1/2, z+1/2	10,75	B3LYP/6-31G(d,p)	0,4	-0,2	-3	0,9	-1,7	-3,4
								E_{subl} (kJ/mol)	-131,50

PGGE

N	Symop	R	Electron Density	E_ele	E_pol	E_dis	E_rep	E_tot	
2	x, y, z	4,7	B3LYP/6-31G(d,p)	-18,5	-4,1	-61	40,8	-50,5	-101
2	x, y, z	7,89	B3LYP/6-31G(d,p)	-2,9	-0,3	-10,5	6,3	-8,6	-17,2
2	x, y, z	8,6	B3LYP/6-31G(d,p)	-1,5	-2,9	-8,2	5,1	-7,7	-15,4
1	-x, -y, -z	9,07	B3LYP/6-31G(d,p)	-3,1	-0,3	-22	11,9	-15,3	-15,3
1	-x, -y, -z	10,75	B3LYP/6-31G(d,p)	-4,2	-4,4	-23,1	14,9	-18,6	-18,6
2	x+1/2, -y+1/2, z+1/2	8,38	B3LYP/6-31G(d,p)	-13,7	-1,2	-18	11,7	-23,8	-47,6
2	x+1/2, -y+1/2, z+1/2	10,45	B3LYP/6-31G(d,p)	-3,3	-3,8	-6,4	4,1	-9,3	-18,6
1	-x, -y, -z	11,21	B3LYP/6-31G(d,p)	-2,2	-1,5	-17,5	9,5	-12,8	-12,8
1	-x, -y, -z	13,02	B3LYP/6-31G(d,p)	-2	0	-7,5	0	-8,7	-8,7
2	x+1/2, -y+1/2, z+1/2	8,69	B3LYP/6-31G(d,p)	5	-2	-14,6	7,8	-4,2	-8,4
								E_{subl} (kJ/mol)	-131,8

EGE (monoclinic, $P2_1$/n)

N	Symop	R	Electron Density	E_ele	E_pol	E_dis	E_rep	E_tot	
2	x, y, z	8,64	B3LYP/6-31G(d,p)	-1,2	-0,5	-7,8	4,5	-5,7	-11,4
2	x, y, z	4,57	B3LYP/6-31G(d,p)	-18,6	-0,3	-59,1	38,4	-47,6	-95,2
1	-x, -y, -z	10,08	B3LYP/6-31G(d,p)	-7,4	-2,2	-26,6	20,5	-19,9	-19,9
2	x+1/2, -y+1/2, z+1/2	10,42	B3LYP/6-31G(d,p)	-3	-0,6	-6,1	3,9	-6,6	-13,2
1	-x, -y, -z	12,46	B3LYP/6-31G(d,p)	-2,2	-0,1	-7	0	-8,5	-8,5
2	x+1/2, -y+1/2, z+1/2	8,41	B3LYP/6-31G(d,p)	-13,9	-9,8	-17,7	11,2	-30,4	-60,8
2	x, y, z	7,86	B3LYP/6-31G(d,p)	-2,7	-0,5	-10,2	5,7	-8,5	-17
1	-x, -y, -z	8,8	B3LYP/6-31G(d,p)	-5,8	-0,9	-24,3	16,5	-17,7	-17,7
2	x+1/2, -y+1/2, z+1/2	8,64	B3LYP/6-31G(d,p)	4,1	-8,4	-15,5	8,9	-9,9	-19,8
1	-x, -y, -z	11,11	B3LYP/6-31G(d,p)	-4,1	-0,4	-15,9	12	-11	-11
								E_{subl} (kJ/mol)	-137,25

EGE (triclinic, *P*-1) major conformer

N	Symop	R	Electron Density	E_ele	E_pol	E_dis	E_rep	E_tot	
2	x, y, z	9,81	B3LYP/6-31G(d,p)	-1,5	-0,4	-8,1	5,1	-5,7	-11,4
1	-x, -y, -z	8,49	B3LYP/6-31G(d,p)	-0,5	-0,4	-11	6	-6,7	-6,7
1	-x, -y, -z	10,17	B3LYP/6-31G(d,p)	-3,9	0	-19,8	14,1	-12,7	-12,7
2	x, y, z	4,8	B3LYP/6-31G(d,p)	-20,1	-3,7	-59,4	38,7	-51,9	-103,8
2	x, y, z	10,32	B3LYP/6-31G(d,p)	-1,7	0	-7	3,3	-5,9	-11,8
1	-x, -y, -z	5,68	B3LYP/6-31G(d,p)	-11,4	-2,1	-32,1	26,6	-25,2	-25,2
1	-x, -y, -z	15,24	B3LYP/6-31G(d,p)	0,1	0	-4,7	0	-4	-4
1	-x, -y, -z	10,73	B3LYP/6-31G(d,p)	0,3	-0,1	-2,1	0,3	-1,4	-1,4
1	-x, -y, -z	9,05	B3LYP/6-31G(d,p)	-20,7	-2,3	-27,8	26,2	-31,6	-31,6
1	-x, -y, -z	8,01	B3LYP/6-31G(d,p)	-7,3	-1,2	-29,6	18,4	-23	-23
1	-x, -y, -z	7,41	B3LYP/6-31G(d,p)	-5,3	-3,5	-20,4	13	-17,9	-17,9

E_{subl} (kJ/mol) -124,75

PGD

Molecule 1

N	Symop	R	Electron Density	E_ele	E_pol	E_dis	E_rep	E_tot	
1	-	7,56	B3LYP/6-31G(d,p)	-9,3	-3,5	-57	34,9	-40,5	-40,5
1	-	7,57	B3LYP/6-31G(d,p)	-12,4	-2,3	-58,6	36,9	-43	-43
1	-	7,55	B3LYP/6-31G(d,p)	-10,2	-3,6	-58,7	37,1	-41,7	-41,7
1	-	7,57	B3LYP/6-31G(d,p)	-12,5	-2,2	-58,7	37	-43,1	-43,1
1	-	13,76	B3LYP/6-31G(d,p)	0	0	-3,3	0	-2,9	-2,9
1	-	13,01	B3LYP/6-31G(d,p)	-2,8	-0,6	-7,4	0	-9,8	-9,8
1	-	11,22	B3LYP/6-31G(d,p)	-0,4	0	-6,6	1,7	-5,1	-5,1
1	-	13,01	B3LYP/6-31G(d,p)	-4,6	-0,6	-7,4	0	-11,8	-11,8
1	-	10,51	B3LYP/6-31G(d,p)	-1,3	-0,1	-9,2	4,6	-6,5	-6,5
1	-x, -y, -z	10,03	B3LYP/6-31G(d,p)	-1,2	0	-13,5	6,1	-9,3	-9,3
2	x, y, z	15,12	B3LYP/6-31G(d,p)	0,2	-0,1	-1,7	0	-1,3	-2,6
2	x, y, z	12,13	B3LYP/6-31G(d,p)	0,6	-0,1	-7,2	0	-5,7	-11,4
2	-x+1/2, y+1/2, -z+1/2	8,45	B3LYP/6-31G(d,p)	-35,4	-10	-26,9	32,7	-48,1	-96,2
2	x, y, z	9,03	B3LYP/6-31G(d,p)	-9,1	-2,7	-18,3	5,1	-24,4	-48,8
1	-x, -y, -z	12,98	B3LYP/6-31G(d,p)	-1,2	-0,1	-6,9	0	-7,4	-7,4
								E_{subl} (kJ/mol)	-190,05

Molecule 2

N	Symop	R	Electron Density	E_ele	E_pol	E_dis	E_rep	E_tot	
1	-	7,56	B3LYP/6-31G(d,p)	-9,3	-3,5	-57	34,9	-40,5	-40,5
2	x, y, z	12,13	B3LYP/6-31G(d,p)	-0,1	-0,1	-7,4	0	-6,6	-13,2

1	-x, -y, -z	10,02	B3LYP/6-31G(d,p)	-1,5	-0,1	-14,4	6	-10,5	-10,5
2	x, y, z	15,12	B3LYP/6-31G(d,p)	-0,6	-0,1	-1,6	0	-2,1	-4,2
2	-x+1/2, y+1/2, -z+1/2	8,45	B3LYP/6-31G(d,p)	-35,9	-10,1	-27,3	33,7	-48,4	-96,8
2	x, y, z	9,03	B3LYP/6-31G(d,p)	-9	-2,7	-18,4	5,2	-24,3	-48,6
2	-x+1/2, y+1/2, -z+1/2	15,31	B3LYP/6-31G(d,p)	0,4	-0,1	-1	0	-0,5	-1
1	-x, -y, -z	15,32	B3LYP/6-31G(d,p)	-0,5	0	-1,6	0	-1,9	-1,9
1	-	7,57	B3LYP/6-31G(d,p)	-12,4	-2,3	-58,6	36,9	-43	-43
1	-	7,55	B3LYP/6-31G(d,p)	-10,2	-3,6	-58,7	37,1	-41,7	-41,7
1	-x, -y, -z	13	B3LYP/6-31G(d,p)	-0,4	0	-2,2	0	-2,3	-2,3
1	-	7,57	B3LYP/6-31G(d,p)	-12,5	-2,2	-58,7	37	-43,1	-43,1
1	-	13,76	B3LYP/6-31G(d,p)	0	0	-3,3	0	-2,9	-2,9
1	-	13,01	B3LYP/6-31G(d,p)	-2,8	-0,6	-7,4	0	-9,8	-9,8
1	-	11,22	B3LYP/6-31G(d,p)	-0,4	0	-6,6	1,7	-5,1	-5,1
1	-	13,01	B3LYP/6-31G(d,p)	-4,6	-0,6	-7,4	0	-11,8	-11,8
1	-	10,51	B3LYP/6-31G(d,p)	-1,3	-0,1	-9,2	4,6	-6,5	-6,5
								E_{subl} (kJ/mol)	-191,45

ED (major conformer)

N	Symop	R	Electron Density	E_ele	E_pol	E_dis	E_rep	E_tot	
1	-	7,51	B3LYP/6-31G(d,p)	-3,6	-3,5	-51,4	28,1	-33,8	-33,8
2	x, y, z	8,97	B3LYP/6-31G(d,p)	-9	-2,6	-17,5	4,6	-23,8	-47,6
1	-	8,28	B3LYP/6-31G(d,p)	-35,1	-8,9	-26,9	31,5	-47,6	-47,6
1	-x, -y, -z	12,34	B3LYP/6-31G(d,p)	-5,3	-0,9	-9,7	0	-14,6	-14,6
1	-	15,1	B3LYP/6-31G(d,p)	0,6	-0,1	-1,1	0	-0,4	-0,4
1	-	7,64	B3LYP/6-31G(d,p)	-12,7	-0,2	-46,6	24,2	-39,1	-39,1
1	-	9,85	B3LYP/6-31G(d,p)	-0,5	-0,1	-10,4	2,8	-7,8	-7,8
1	-	7,62	B3LYP/6-31G(d,p)	-6	-2,1	-52	30,1	-34,5	-34,5
2	x, y, z	12,47	B3LYP/6-31G(d,p)	-0,9	-0,2	-6,9	0	-7,1	-14,2
1	-x, -y, -z	13,26	B3LYP/6-31G(d,p)	-0,1	-0,7	-7	0	-6,7	-6,7
1	-	7,96	B3LYP/6-31G(d,p)	-13,1	-5,3	-44,7	26,3	-40,5	-40,5
1	-	12,87	B3LYP/6-31G(d,p)	-0,9	-0,1	-5,8	0	-6	-6
1	-x, -y, -z	9,67	B3LYP/6-31G(d,p)	-2,1	-4,1	-13	5,5	-13,1	-13,1
1	-	8,37	B3LYP/6-31G(d,p)	-33,9	-2,1	-26	29,4	-41,9	-41,9
1	-	15,26	B3LYP/6-31G(d,p)	0,7	-0,1	-1	0	-0,3	-0,3
								E_{subl} (kJ/mol)	-174,05

MPGD

N	Symop	R	Electron Density	E_ele	E_pol	E_dis	E_rep	E_tot	
2	-x+1/2, y+1/2, z	7,56	B3LYP/6-31G(d,p)	-15,2	-0,1	-46,2	27	-39,6	-79,2
2	x+1/2, y+1/2, -z+1/2	10,33	B3LYP/6-31G(d,p)	-38,3	-0,4	-15,2	53,7	-20,9	-41,8
2	-x+1/2, y+1/2, z	9,29	B3LYP/6-31G(d,p)	-5,3	-1,9	-24,8	13,7	-20,2	-40,4
2	x, y, z	8,83	B3LYP/6-31G(d,p)	-8,3	-1,5	-42,7	26	-30,9	-61,8
1	-x, -y, -z	9,95	B3LYP/6-31G(d,p)	-1,9	-0,7	-23,1	7,4	-18	-18
2	x+1/2, -y+1/2, -z	11,63	B3LYP/6-31G(d,p)	-0,5	0	-4	1,3	-3,2	-6,4
2	-x, y, -z+1/2	10,73	B3LYP/6-31G(d,p)	-3,5	-1	-10,6	4,3	-11	-22
2	x+1/2, -y+1/2, -z	14,22	B3LYP/6-31G(d,p)	-2,7	-0,3	-6,4	0	-8,6	-17,2
1	-x, y, -z+1/2	6,1	B3LYP/6-31G(d,p)	-82,8	-19,9	-60,3	121,1	-80	-80
1	-x, -y, -z	10,5	B3LYP/6-31G(d,p)	-1,2	-0,1	-8,7	6,1	-5,1	-5,1
								E_{subl} (kJ/mol)	-185,95

References

(1) Feghali, E.; van de Pas, D. J.; Torr, K. M. Toward Bio-Based Epoxy Thermoset Polymers from Depolymerized Native Lignins Produced at the Pilot Scale. *Biomacromolecules* **2020**, *21* (4), 1548–1559. https://doi.org/10.1021/acs.biomac.0c00108.

(2) Quinsaat, J. E. Q.; Feghali, E.; van de Pas, D. J.; Vendamme, R.; Torr, K. M. Preparation of Biobased Nonisocyanate Polyurethane/Epoxy Thermoset Materials Using Depolymerized Native Lignin. *Biomacromolecules* **2022**, *23* (11), 4562–4573. https://doi.org/10.1021/acs.biomac.2c00706.

(3) van de Pas, D. J.; Nanayakkara, B.; Suckling, I. D.; Torr, K. M. Comparison of Hydrogenolysis with Thioacidolysis for Lignin Structural Analysis. *Holzforschung* **2014**, *68* (2), 151–155. https://doi.org/10.1515/hf-2013-0075.

(4) Gracia-Vitoria, J.; Rubens, M.; Feghali, E.; Adriaensens, P.; Vanbroekhoven, K.; Vendamme, R. Low-Field Benchtop versus High-Field NMR for Routine 31P Analysis of Lignin, a Comparative Study. *Ind Crops Prod* **2022**, *176*, 114405. https://doi.org/10.1016/j.indcrop.2021.114405.

(5) Granata, A.; Argyropoulos, D. S. 2-Chloro-4,4,5,5-Tetramethyl-1,3,2-Dioxaphospholane, a Reagent for the Accurate Determination of the Uncondensed and Condensed Phenolic Moieties in Lignins. *J Agric Food Chem* **1995**, *43* (6), 1538–1544. https://doi.org/10.1021/jf00054a023.

(6) Meng, X.; Crestini, C.; Ben, H.; Hao, N.; Pu, Y.; Ragauskas, A. J.; Argyropoulos, D. S. Determination of Hydroxyl Groups in Biorefinery Resources via Quantitative 31P NMR Spectroscopy. *Nat Protoc* **2019**, *14* (9), 2627–2647. https://doi.org/10.1038/s41596-019-0191-1.

(7) Rigaku Oxford Diffraction, CrysAlisPro Software System, Version 1.171.Xx.Xx; Rigaku Corporation: Oxford, UK, 2018.

(8) Dolomanov, O. V.; Bourhis, L. J.; Gildea, R. J.; Howard, J. A. K.; Puschmann, H. OLEX2: A Complete Structure Solution, Refinement and Analysis Program. *J Appl Crystallogr* **2009**, *42*, 339–341. https://doi.org/10.1107/S0021889808042726.

(9) Sheldrick, G. M. SHELXT – Integrated Space-Group and Crystal-Structure Determination. *Acta Crystallogr A Found Adv* **2015**, *71* (1), 3–8. https://doi.org/10.1107/S2053273314026370.

(10) Sheldrick, G. M. Crystal Structure Refinement with SHELXL. *Acta Crystallogr C Struct Chem* **2015**, *71* (1), 3–8. https://doi.org/10.1107/S2053229614024218.

(11) CrystalClear 2.1: Rigaku, CrystalClear SM-Expert v2.1 B32, April 12, 2014.

(12) Minor, W.; Cymborowski, M.; Otwinowski, Z.; Chruszcz, M. HKL-3000: The Integration of Data Reduction and Structure Solution – from Diffraction Images to an Initial Model in Minutes. *Acta Crystallogr D Biol Crystallogr* **2006**, *62* (8), 859–866. https://doi.org/10.1107/S0907444906019949.

(13) Hübschle, C. B.; Sheldrick, G. M.; Dittrich, B. ShelXle: A Qt Graphical User Interface for SHELXL. *J Appl Crystallogr* **2011**, *44* (6), 1281–1284. https://doi.org/10.1107/S0021889811043202.

(14) Spackman, P. R.; Turner, M. J.; McKinnon, J. J.; Wolff, S. K.; Grimwood, D. J.; Jayatilaka, D.; Spackman, M. A. CrystalExplorer: A Program for Hirshfeld Surface Analysis, Visualization and Quantitative Analysis of Molecular Crystals. *J Appl Crystallogr* **2021**, *54* (3), 1006–1011. https://doi.org/10.1107/S1600576721002910.

(15) Mackenzie, C. F.; Spackman, P. R.; Jayatilaka, D.; Spackman, M. A. CrystalExplorer Model Energies and Energy Frameworks: Extension to Metal Coordination Compounds, Organic Salts, Solvates and Open-Shell Systems. *IUCrJ* **2017**, *4* (5), 575–587. https://doi.org/10.1107/S205225251700848X.

(16) Jayatilaka, D.; Grimwood, D. J. Tonto: A Fortran Based Object-Oriented System for Quantum Chemistry and Crystallography. In *Computational Science — ICCS 2003*; Sloot, P. M. A., Abramson, D., Bogdanov, A. V., Gorbachev, Y. E., Dongarra, J. J., Zomaya, A. Y., Eds.; Springer: Berlin, Heidelberg, 2003; Vol. 2660, pp 142–151. https://doi.org/10.1007/3-540-44864-0_15.

(17) Quinsaat, J. E. Q.; Feghali, E.; van de Pas, D. J.; Vendamme, R.; Torr, K. M. Preparation of Mechanically Robust Bio-Based Polyurethane Foams Using Depolymerized Native Lignin. *ACS Appl Polym Mater* **2021**, *3* (11), 5845–5856. https://doi.org/10.1021/acsapm.1c01081.

(18) Pepper, J. M.; Sundaram, G. S.; Dyson, G. Lignin and Related Compounds. III. An Improved Synthesis of 3-(4-Hydroxy-3-Methoxyphenyl)-1-Propanol and 3-(4-Hydroxy-3,5-Dimethoxyphenyl)-1-Propanol. *Can J Chem* **1971**, *49* (20), 3394–3395. https://doi.org/10.1139/v71-564.

(19) Hemelaere, R.; Carreaux, F.; Carboni, B. A Diastereoselective Route to Trans-2-Aryl-2,3-Dihydrobenzofurans through Sequential Cross-Metathesis/Isomerization/Allylboration Reactions: Synthesis of Bioactive Neolignans. *European J Org Chem* **2015**, *2015* (11), 2470–2481. https://doi.org/10.1002/ejoc.201500019.

(20) Rubens, M.; Falireas, P.; Vanbroekhoven, K.; Van Hecke, W.; Kaya, G. E.; Baytekin, B.; Vendamme, R. Molecular Design of Lignin-Derived Side-Chain Phenolic Polymers toward Functional Radical Scavenging Materials with Antioxidant and Antistatic Properties. *Biomacromolecules* **2023**. https://doi.org/10.1021/acs.biomac.3c00275.

(21) Lahive, C. W.; Kamer, P. C. J.; Lancefield, C. S.; Deuss, P. J. An Introduction to Model Compounds of Lignin Linking Motifs; Synthesis and Selection Considerations for Reactivity Studies. *ChemSusChem* **2020**, *13* (17), 4238–4265. https://doi.org/10.1002/cssc.202000989.

(22) Ogata, M.; Hoshi, M.; Urano, S.; Endo, T. Antioxidant Activity of Eugenol and Related Monomeric and Dimeric Compounds. *Chem Pharm Bull (Tokyo)* **2000**, *48* (10), 1467–1469. https://doi.org/10.1248/cpb.48.1467.

(23) Audrey Llevot. Dimères d'acides Résiniques et de Dérivés de La Lignine: Nouveaux Précurseurs Pour La Synthèse de Polymères Bio-Sourcés. Polymères., Université de Bordeaux, 2014.

(24) Savonnet, E.; Grau, E.; Grelier, S.; Defoort, B.; Cramail, H. Divanillin-Based Epoxy Precursors as DGEBA Substitutes for Biobased Epoxy Thermosets. *ACS Sustain Chem Eng* **2018**, *6* (8), 11008–11017. https://doi.org/10.1021/acssuschemeng.8b02419.

(25) Llevot, A.; Grau, E.; Carlotti, S.; Grelier, S.; Cramail, H. Selective Laccase-Catalyzed Dimerization of Phenolic Compounds Derived from Lignin: Towards Original Symmetrical Bio-Based (Bis) Aromatic Monomers. *J Mol Catal B Enzym* **2016**, *125*, 34–41. https://doi.org/10.1016/j.molcatb.2015.12.006.

(26) Llevot, A.; Grau, E.; Carlotti, S.; Grelier, S.; Cramail, H. Renewable (Semi)Aromatic Polyesters from Symmetrical Vanillin-Based Dimers. *Polym Chem* **2015**, *6* (33), 6058–6066. https://doi.org/10.1039/C5PY00824G.

(27) *Dieugenol Item No. 27557*. https://www.caymanchem.com/product/27557 (accessed 2023-07-25).

(28) Zhang, D.; Jin, S.; Wan, J.; Wang, J.; Li, Y.; Jin, P.; Hu, D. A Dieugenol-Based Epoxy Monomer with High Bio-Based Content, Low Viscosity and Low Flammability. *Mater Today Commun* **2021**, *29*, 102846. https://doi.org/10.1016/j.mtcomm.2021.102846.

(29) *Dieugenol D938640*. https://www.trc-canada.com/product-detail/?D938640 (accessed 2023-07-25).

(30) Snyder, R. L.; Lidston, C. A. L.; De Hoe, G. X.; Parvulescu, M. J. S.; Hillmyer, M. A.; Coates, G. W. Mechanically Robust and Reprocessable Imine Exchange Networks from Modular Polyester Pre-Polymers. *Polym Chem* **2020**, *11* (33), 5346–5355. https://doi.org/10.1039/C9PY01957J.

(31) Liu, B.; Chen, J.; Liu, N.; Ding, H.; Wu, X.; Dai, B.; Kim, I. Bio-Based Polyesters Synthesized by Ring-Opening Copolymerizations of Eugenyl Glycidyl Ether and Cyclic Anhydrides Using a Binuclear [OSSO]CrCl Complex. *Green Chemistry* **2020**, *22* (17), 5742–5750. https://doi.org/10.1039/d0gc00469c.

(32) Cattey, H.; Boni, G.; Pourchet, S.; Plasseraud, L. Crystal Structure of the Monoglycidyl Ether of Isoeugenol. *Acta Crystallogr E Crystallogr Commun* **2022**, *78* (10), 1052–1055. https://doi.org/10.1107/S2056989022009264.

(33) Caillol, S.; Auvergne, R.; Manseri, A.; Boutevin, G.; Boutevin, B.; Grimaldi, M.; Balaguer, P. Understanding Glycidylation Reaction for the Formation of Pure Mono, Diglycidyl and Dual Monomers as Glycidyl Methacrylate of Vanillyl Alcohol. *J Appl Polym Sci* **2023**, *140* (10). https://doi.org/10.1002/app.53596.

(34) Koelewijn, S.-F.; Van den Bosch, S.; Renders, T.; Schutyser, W.; Lagrain, B.; Smet, M.; Thomas, J.; Dehaen, W.; Van Puyvelde, P.; Witters, H.; Sels, B. F. Sustainable Bisphenols from Renewable Softwood Lignin Feedstock for Polycarbonates and Cyanate Ester Resins. *Green Chemistry* **2017**, *19* (11), 2561–2570. https://doi.org/10.1039/C7GC00776K.

(35) Liu, J.; Zhang, X.; Liu, S.; Lei, C. Allyl-Modified Epoxy Networks That Break and Reorganize at High Temperatures to Avoid T Reduction. *Chemical Engineering Journal* **2023**, *455*, 140674. https://doi.org/10.1016/j.cej.2022.140674.

(36) Duann, Y.-F.; Liu, T.-M.; Cheng, K.-C.; Su, W.-F. Thermal Stability of Some Naphthalene- and Phenyl-Based Epoxy Resins. *Polym Degrad Stab* **2004**, *84* (2), 305–310. https://doi.org/10.1016/j.polymdegradstab.2004.01.016.

(37) Vigier, J.; François, C.; Pourchet, S.; Boni, G.; Plasseraud, L.; Placet, V.; Fontaine, S.; Cattey, H. Crystal Structure of the Diglycidyl Ether of Eugenol. *Acta Crystallogr E Crystallogr Commun* **2017**, *73* (5), 694–697. https://doi.org/10.1107/S2056989017005370.

(38) Fang, Z.; Nikafshar, S.; Hegg, E. L.; Nejad, M. Biobased Divanillin As a Precursor for Formulating Biobased Epoxy Resin. *ACS Sustain Chem Eng* **2020**, *8* (24), 9095–9103. https://doi.org/10.1021/acssuschemeng.0c02351.

(39) de Souza, E. C.; Romero-Ortega, M.; Olivo, H. F. Lipase-Mediated Selective Acetylation of Primary Alcohols in Ethyl Acetate. *Tetrahedron Lett* **2018**, *59* (3), 287–290. https://doi.org/10.1016/j.tetlet.2017.12.041.
